# Camelids and Cattle Are Dead-End Hosts for Peste-des-Petits-Ruminants Virus

**DOI:** 10.3390/v11121133

**Published:** 2019-12-08

**Authors:** Claudia Schulz, Christine Fast, Ulrich Wernery, Jörg Kinne, Sunitha Joseph, Kore Schlottau, Maria Jenckel, Dirk Höper, Nissy Annie Georgy Patteril, Ginu Syriac, Bernd Hoffmann, Martin Beer

**Affiliations:** 1 Institute of Diagnostic Virology, Friedrich-Loeffler-Institut, 17493 Greifswald-Insel Riems, Germany; kore.schlottau@fli.de (K.S.); maria.jenckel@csiro.au (M.J.); dirk.hoeper@fli.de (D.H.); bernd.hoffmann@fli.de (B.H.); martin.beer@fli.de (M.B.); 2 Institute of Novel and Emerging Infectious Diseases, Friedrich-Loeffler-Institut, 17493 Greifswald-Insel Riems, Germany; christine.fast@fli.de; 3 Central Veterinary Research Institute, P.O. Box 597 Dubai, UAE; cvrl@cvrl.ae (U.W.);

**Keywords:** Peste-des-petits-ruminants virus, small ruminant morbillivirus, transmission, experimental infection, Artiodactyla, cattle, camel, alpaca, llama, dromedary camel

## Abstract

Peste-des-petits-ruminants virus (PPRV) causes a severe respiratory disease in small ruminants. The possible impact of different atypical host species in the spread and planed worldwide eradication of PPRV remains to be clarified. Recent transmission trials with the virulent PPRV lineage IV (LIV)-strain Kurdistan/2011 revealed that pigs and wild boar are possible sources of PPRV-infection. We therefore investigated the role of cattle, llamas, alpacas, and dromedary camels in transmission trials using the Kurdistan/2011 strain for intranasal infection and integrated a literature review for a proper evaluation of their host traits and role in PPRV-transmission. Cattle and camelids developed no clinical signs, no viremia, shed no or only low PPRV-RNA loads in swab samples and did not transmit any PPRV to the contact animals. The distribution of PPRV-RNA or antigen in lymphoid organs was similar in cattle and camelids although generally lower compared to suids and small ruminants. In the typical small ruminant hosts, the tissue tropism, pathogenesis and disease expression after PPRV-infection is associated with infection of immune and epithelial cells via SLAM and nectin-4 receptors, respectively. We therefore suggest a different pathogenesis in cattle and camelids and both as dead-end hosts for PPRV.

## 1. Introduction

Peste-des-petits-ruminants (PPR) is a highly contagious notifiable transboundary disease caused by PPR virus (PPRV, species: *small ruminant morbillivirus* [1]) that mainly affects small ruminants. Since the worldwide eradication of the closely related rinderpest virus (RPV, species: *rinderpest morbillivirus*) in 2011, PPRV has spread considerably across African and Asian countries, in particular lineage (L)IV of the four known PPRV lineages (LI to IV) [2]. Clinical signs in small ruminants, particularly in goats, include fever, erosions of mucosal membranes, nasal and ocular discharge, respiratory distress, diarrhea, and death [3,4]. In sheep and other wild or domestic species within the Artiodactyla order (even-toed ungulates), disease expression associated with PPRV-infection may vary between subclinical infection to severe clinical signs and death, similar to goats [5]. The high morbidity and mortality rates of up to 100% in small ruminants threatens the livelihood of the poorest people that depend on small ruminant production and results in considerable economical losses of affected countries around the globe. Therefore, the eradication of PPRV is targeted for 2030 [2,6]. For RPV, reservoir hosts including various wildlife species had been determined [7,8], while for the spread of PPRV the possible role of atypical hosts of other domestic and wild artiodactyls still remains largely unknown [5]. The identification of reservoir hosts that may contract the disease particularly by silent spread over large distances and across borders without expression of obvious clinical signs is of major concern. For example, sheep subclinically infected with PPRV are a known possible source of silent PPRV-spread [5,9]. PPRV RNA, antigen or infectious virus was detected in blood or tissue samples from different wild animal species [5,10], camels [11,12], buffaloes [13], and cattle [14], but no transmission to susceptible contact animals or shedding of infectious virus has so far been reported in any sound study for these Artiodactyla species. Additionally, due to multiple disease outbreaks in camels, which were associated with PPRV-infection with any of the lineages LII, LIII [15], or LIV, PPR has been discussed an emerging disease in camels [12,16,17,18]. El-Hakim [19] reports transmission of a PPRV LI strain from camels to goats, but the study design does not preclude natural infection with PPRV or other pathogens causing PPR-like clinical signs, because infection route, biosafety, and sanitary measures have not been described. 

Similar to cattle, pigs were previously considered dead-end hosts for PPRV [18]. However, PPRV transmission from pigs to a contact pig and a contact goat, and the excretion of infectious PPRV by pigs and wild boar after experimental intranasal infection with the virulent PPRV LIV strain Kurdistan/2011 showed recently that suids may indeed act as a potential source of PPRV infection [9]. Similarly, transmission of RPV to contact cattle was recorded for pigs [20], but not for camels experimentally infected with RPV, despite of using different inoculation routes (intravenous (i.v.), subcutaneous (s.c.), contact to RPV-infected cattle) and the detection of infectious PPRV in the blood of some camels [21]. 

Taken together the unclear host traits of various species indicates that single or multiple species together may be a potential additional source of PPRV-infection depending on various host, virus and/or environmental factors in an ecosystem. For example, stress, concurrent infections, abundance, density and behavior/interaction of host species, virulence, shedding patterns and survival of a virus strain, climate, and anthropogenic practice (e.g., transhumance, land use) [9,22,23,24,25,26,27]. In general, the basic reproductive number (*R*_0_; expected number of secondary infections caused by a typical infected individual in a susceptible population) determines whether a pathogen can cause (i) a major epidemic by sustained transmission (*R*_0_ > 1; maintenance/reservoir host), (ii) a self-limiting outbreaks (0 < *R*_0_ < 1; spillover/recipient host), or (iii) a single event of transmission (*R*_0_ = 0; dead-end spillover/recipient host) [26]. For PPRV, goats and sheep are recognized reservoir hosts that may transmit PPRV, show typical PPR disease and a similar pathogenesis after PPRV infection [2,3,4,9,28]. The host traits of other species susceptible to PPRV-infection according to serological or virological evidence is still under investigation or unknown. We therefore suggest the terms “typical” hosts for domestic small ruminants and “atypical” hosts for other susceptible host species of PPRV, similar to Bataille, et al. [29] and Abdullah, et al. [30]. 

Variations in receptor specificity influence host range, tissue tropism, pathogenicity and transmissibility of different viruses [31]. Morbilliviruses are negative-sense single stranded RNA viruses. The six transcriptional units of the PPRV-genome encode for six structural proteins: Nucleoprotein (N), phosphoprotein (P), matrix protein (M), the fusion (F) and hemagglutinin (H) membrane glycoproteins, and the polymerase (L). The P gene also encodes for the two non-structural proteins C and V [32,33]. Similar to PPRV, a wide host range for the other animal morbilliviruses (canine distemper virus (CDV), cetacean morbillivirus (CeMV) and RPV that infect respectively carnivores, cetaceans and artiodactyls) has been reported [7,8,32,34,35,36,37]. Attachment of morbilliviruses to cell surfaces is mediated via their H glycoprotein by direct protein–protein interaction with the two major natural host receptors involved in morbillivirus infection: CD150 (signaling lymphocyte activation molecule, SLAM) and nectin-4 [32]. Fusion activity of the F protein allows virus entry into the cell cytoplasm [32]. SLAM receptors are present on activated lymphocytes, macrophages and dendritic cells (DC) and lead to systemic morbillivirus infection, while the infection of epithelial cells via nectin-4 receptors plays an important role in disease development and virus transmission [37,38,39,40]. The tropism of morbilliviruses for immune cells and lymphoid organs may lead to immune suppression and a higher susceptibility to secondary infections and disease expression [9,39]. The limited inherent capacity for antigenic variation of morbillivirus glycoproteins [37], and the high conservation of the SLAM and nectin-4 receptors across mammalian species facilitate cross-species transmission of animal morbilliviruses between related mammalian species [35,38]. 

We aimed to scrutinize the contradictory reports of PPRV-infection associated with and without clinical signs in camelids and cattle and to elucidate their potential role in the epidemiology and eradication of PPRV. Furthermore, PPRV-infection of South American camelids (SAC) has never been reported. Accordingly, in light of our results from three independent transmission experiments with cattle, SAC and dromedaries using the PPRV/Kurdistan/2011 strain virulent in small ruminants and suids [3,9,41], we conducted a comprehensive literature review. The results suggest cattle and camelids are dead-end hosts for PPRV.

## 2. Materials and Methods

### 2.1. Virus

The small ruminant morbillivirus lineage IV (PPRV-LIV) strain Kurdistan/2011 was isolated on CHS-20 (goat-SLAM) cells [42] from a lung sample from a wild goat (bezoar ibex, Capra aegagrus) in Iraq [3,9,41] (lab submission no. BH15/11-5; Accession no. JF969755.1, KF648288, KF648287.1). The virus was passaged twice on vero.dog.slam.tag cells [43] (10^4.83 TCID50/mL, quantification cycle (Cq) value 15.01) and this virus stock (PPRV Kurdistan/2011/BH15-11_5/1CHS/2VDS 11/06/14) was used for all transmission trials as described previously [9]. 

The full-coding genome of the PPRV-LIV strain Kurdistan/2011 was obtained by next generation sequencing according to [44,45] and published in GenBank (Accession no. MK408669). The highest identity (98%) and alignment score of the Kurdistan/2011 strain was found with PPRV LIV strain Turkey/2000 (Accession no. AJ849636) (BLASTN 2.8.1+; http://blast.ncbi.nlm.nih.gov/Blast.cgi). 

### 2.2. Animals and Study Design

**Figure 1:**
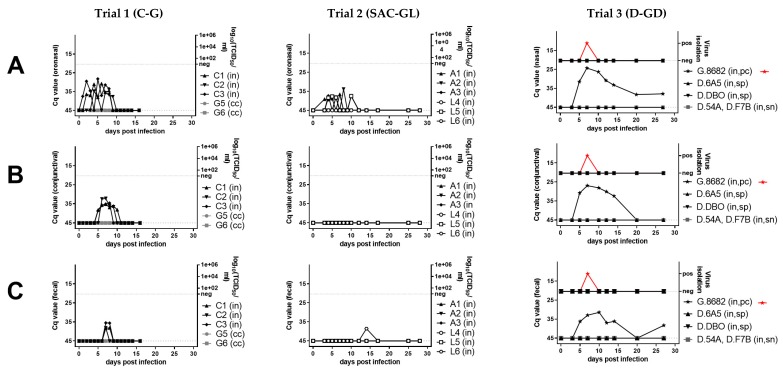

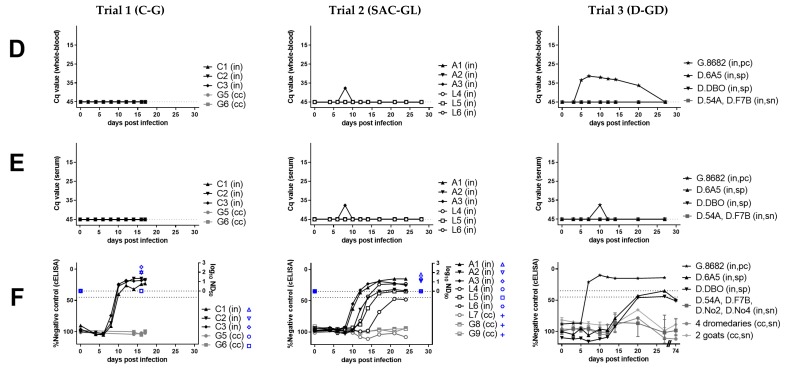

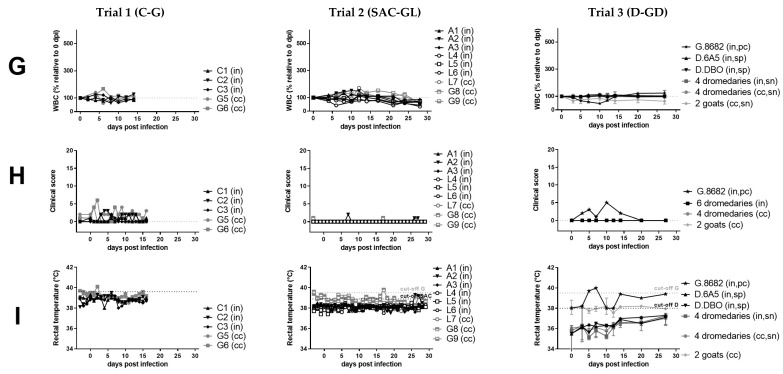
Virological (**A**–**E**), serological (**F**), hematological (**G**), and clinical (**H**–**I**) results of peste-des-petits-ruminants virus (PPRV) transmission trials after intranasal experimental infection (in) of three cattle (C, trial 1), six South American camelids (SAC, trial 2) (three alpaca (A) and three lama (L)), six dromedaries (D) and one positive control (pc) goat (G) (trial 3) with PPRV lineage IV strain Kurdistan/2011 [41]. Panels belonging to anyone of the three trials are shown from top to bottom (trials 1A–I, trial 2 A–I, trial 3A–I). Panels from left to right show samples by trial analyzed with the same methods. (**A**) PPRV or PPRV-RNA loads in oronasal swabs, (**B**) PPRV or PPRV-RNA loads in conjunctival swabs, (**C**) PPRV or PPRV-RNA loads in fecal swabs, (**D**) PPRV-RNA loads in whole-blood, (**E**) PPRV-RNA loads in serum, (**F**) PPRV antibody levels or neutralizing antibody titers in serum, (**G**) proportion of white-blood-cells (WBC) relative to day 0 (before infection), (**H**) clinical score values, (**I**) rectal body temperature values. For samples from trials that were additionally analyzed by virus titration (TCID_50_/mL), virus isolation (red symbols) or neutralization test (ND_50_) (blue symbols) the respective analysis is given on the y-axis of the graphs. PPRV could not be isolated from any of the cattle or camelids, but PPRV was isolated from the positive control (pc) goat (G.8682) (red symbols) of the dromedary trial. In the panels f and g of trial 3, individual results were presented for dromedaries that seroconverted (seropositive, sp) and the positive control (pc)-goat, while median and range values of dromedaries (*n* = 4) that remained refractory (seronegative, sn) to intranasal (in) PPRV-inoculation and of contact control (cc) dromedaries (*n* = 4) and goats (*n* = 2) are shown to allow a clearer overview of the data. Cq, quantitative cycle value of PPRV-RNA quantified by real-time quantitative reverse transcription-PCR (RT-qPCR) with the PPRV-assay of Batten et al. 2011 [50]; TCID_50_/ml, 50% tissue culture infective dose obtained by virus titration assay using vero.dog.SLAM.tag cells [43] or CHS-20 (goat-SLAM) cells [42] (both cell lines show a similar sensitivity for virus isolation from different animal species [9]; cELISA, competition ELISA (IDvet); ND_50_, virus neutralization by PPRV antibodies in 50% of the replicates.

All animal experiments conducted in Germany were approved by the competent authorities of the Federal State of Mecklenburg–Western Pomerania and are in accordance with European legislation concerning animal welfare in particular directive 2010/63/EU. The experimental protocol of the trial with dromedaries was reviewed and approved by the ethics commission at the Central Veterinary Research Laboratory (Dubai, United Arab Emirates). To investigate whether PPRV-infected cattle and camelids may transmit infectious PPRV to PPRV-naïve contact goats or animals of the same species, PPRV transmission trials were conducted with cattle, South American camelids (SAC) and dromedaries (Table 1). Prior to experimental PPRV-infection, all animals were confirmed to be free of a previous PPRV infection with cELISA, they were checked by veterinarians that declared them clinically healthy and naïve to PPRV (trials 1 to 3; Figure 1), and animals of trials 1 and 2 were dewormed. 

PPRV transmission trials with cattle (trial 1) and SAC (trial 2) were conducted separately in the containment facility of the Friedrich-Loeffler-Institut, Isle of Riems, Germany. The cattle (C1–C3), alpacas (A1–A3), and llamas (L4–L6) (Table 1) were intranasally (i.n.) infected with 2 mL of approximately 10^4.5 TCID50/mL (1 mL in each nostril) using nasal atomizers (LMA MADTM 100, Wolfram Droh GmbH, Mainz, Germany). Two to three days after experimental infection (dpi), contact-control animals were added to the same enclosures of the cattle or SAC (see details in Table 1) and fed from the same feeding trough. 

The PPRV transmission trial with ten dromedaries (trial 3) was conducted at the Central Veterinary Research Institute, Dubai, United Arab Emirates. Three young (D.6A5, D.DBO, D.54A) and three old (D.F7B, D.No2, D.No4) dromedaries (D) and one positive control (pc)-goat (G.8682) were i.n. infected using Pocket Nebulizer, Micro A-I-R, Omron, Germany (D.6A5, D.DBO, D.No4, pc-goat) or Pasteur pipette (D.54A, D.F7B, D.No2). The three experimentally infected young adult dromedaries and two young adult contact control dromedaries (D.204, D.O5E) were kept in the same enclosure outdoor (pen 1). Two contact control goats (G.9499, G.9500) were kept in a small enclosure separated by a fence from pen 1 (to avoid serious injury of the goats by aggressive behavior of the dromedaries), which allowed direct nasal contact between dromedaries and goats. In pen 2, three experimentally infected old dromedaries and two old contact control dromedaries (D.No1, D.No6) were kept. The pc-goat was completely separated from the other two pens indoor (pen 3). 

For all animals, rectal body temperature and clinical score was recorded daily (trials 1 and 2) or in regular intervals (trial 3) from a few days before experimental infection until the end of the experiments (Figure 1H,I). Clinical signs were evaluated using the clinical score sheet published by Pope, et al. [4] for small ruminants. For cattle [46] and camelids [47] the score sheet was adapted (Table S1a respectively Table S1b). Clinical signs were scored cumulatively (see further details in Table S1a,b) and graded as follows: None obvious (clinical score (CS) 0), mild (CS 1 to 4), moderate (CS 5 to 9), and severe (CS ≥ 10). The clinical score of the cattle and SAC was assessed by the same veterinarian, the dromedaries and goats of trial 3 by multiple veterinarians. Oronasal (cattle, SAC) or nasal (dromedaries, pc-goat), conjunctival and fecal swab samples as well as serum and EDTA-treated whole-blood samples (all animals) were collected at regular intervals (Figure 1A–E). Swab sample collection, processing and storage was conducted according to Schulz, et al. [9]. 

### 2.3. Serological and Hematological Analyses

Serological and hematological analyses were generally conducted as described previously [9]. Briefly, serum samples collected at regular intervals (Figure 1F,G) were tested with competitive ELISA (ID Screen^®^ PPR competition, ID.vet, France) [48] and selected samples with a standard microneutralization test against the PPRV Kurdistan/2011 isolate used for experimental infection. Low, moderate and high neutralizing antibody (NAb) titers were distinguished according to [9] (<1.5, 1.5, to 2.5 respectively >2.5 log10 ND50 (ND50: virus neutralization in 50% of the replicates [49])). Leucocytes from cattle, SAC and the respective contact animals were counted in a Neubauer counting chamber after lysis of erythrocytes by diluting whole blood 1:100 in 3% acetic acid. The full blood count from dromedary and the pc-goat whole blood samples (trial 3) was automatically analyzed using a hematological analyzer (CELL-DYN 3700, Abbott, IL, USA).

### 2.4. Virological Analyses

Virological analyses of serum, whole-blood, swab samples collected at regular intervals (Figure 1A–E) and of tissue samples were conducted as previously described [9]. Briefly, we used NucleoMagVET kit (Macherey-Nagel, Düren, Germany) on a KingFisher platform (KingFisher Flex, Thermo Fisher Scientific, Schwerte, Germany) for extraction and the real-time quantitative reverse transcription-PCR (RT-qPCR) assay of Batten et al. [50] for PPRV-RNA quantification. An internal control system was used to verify proper RNA extraction and detection as described [51], as well as negative and positive controls. PPRV-RNA loads were differentiated to high (quantitative cycle value (Cq) 18-24.999), moderate (Cq 25-29.999), low (Cq 30-34.999), weak (Cq 35-42), and negative (no Cq). All samples collected in trials 1 and 2 were analyzed by RT-qPCR. Of the 6 experimentally PPRV-inoculated dromedaries, RT-PCR analysis was conducted with samples from 4 dromedaries that showed seropositive results (D.6A5, D.DBO) by cELISA or a slight transient rise in antibody levels below the cut off at 20 dpi (D.54A, D.F7B) (Figure 1F). 

PCR-positive samples from SAC and cattle were subjected to endpoint dilution assays for virus quantification using Vero.dog.slam.tag (green monkey) (VDS) cells [43] and CHS-20 (monkey CV1) (CHS-20) cells [42] in parallel as described [9]. All swab samples collected from all dromedaries and goats of trial 3 were proceeded to virus isolation on CHS-20 cells. 

An anti-PPRV-nucleoprotein (Np) purified monoclonal mouse antibody (Mab anti-PPR, concentration 1 mg/mL, 50% Glycerin, ID.vet) and Alexa 488 (goat anti-mouse) fluorophore were used for indirect immunofluorescence staining.

### 2.5. Pathological Analyses

Selected animals were examined post-mortem for gross pathological lesions. Dromedaries were not euthanized at the end of the trial. Representative tissue samples of 24 (PCR) and 19 (IHC) organs from the three cattle as well as 29 (PCR) and 24 (IHC) organs from the seven SAC and ten (PCR) organs from the one pc-goat were processed for RT-qPCR (PCR) and/or for immunohistochemical (IHC) (see Table 2 for results of selected samples) and histopathological (HP) analyses, respectively, according to [9]. Briefly, before HP and IHC examination, tissue samples were fixed in 10% buffered formalin (4% solution of formaldehyde) and embedded in paraffin. For IHC, a primary anti-PPRV-Np purified monoclonal mouse antibody (Mab anti-PPR, stock concentration 1 mg/mL, 50% Glycerin, ID.vet) was used in a dilution of 1:100 in Tris-buffered saline. Subsequently, 3 μm sections were cut, deparaffinised and rehydrated. Endogenous peroxidase was blocked by 3% H_2_O_2_/methanol incubation and antigen was retrieved with high temperature in the microwave in citrate buffer (pH 6.0, 20 min at 600 W). As secondary antibody Mouse Envision HRP (Dako Diagnostics, Dako Deutschland GmbH, 22047 Hamburg, Germany) and as a substrate diaminobenzidine were used. All tissues (for a summary see Table 2) were investigated with a light microscope and scored according to the proportion of positive cells in the different samples: from negative (0% positive cells), weak (1–5% positive cells), mild (6–25% positive cells), moderate (26–75% positive cells) to severe (>75% positive cells).

### 2.6. Comparison of Performance Characteristics of Different Virological Methods

Four different virological diagnostic methods were compared for their performance characteristic to detect a PPRV infection in (*n* = 20, whole blood, serum, swabs, tissue) from cattle and SAC (Table 3) as described previously [9] using RT-qPCR, virus isolation (VDS and CHS-20 cells) as well as antigen-capture ELISA (ag-ELISA) and lateral flow device (LFD) (see details in Table 3). Only samples positive by PPRV-PCR assay were included in this evaluation.

## 3. Results

### 3.1. Clinical and Hematological Results Suggest Resistance of Camelids and Cattle to PPR-Induced Disease

We observed no or only transient mild and unspecific clinical signs in all experimentally PPRV-infected dromedaries, SAC and cattle (peak clinical scores of 0, 1, or 2 to 3, respectively, Figure 1H), while the positive control (pc)-goat showed mild to moderate clinical signs with a peak clinical score of 5, similar as reported previously for goats [4,9]. No fever was recorded in any animal, except the pc-goat (from 5 to 7 dpi). In camelids, no clinical signs were observed, except in one alpaca (A2) that showed a mild, paste-like diarrhea at 7 dpi (PPRV-RNA negative). Mild unspecific clinical signs in the three heifers included transient mild ocular or nasal discharge at single or a few consecutive days between 3 and 13 dpi, prolonged recumbency in two cattle (C1 and C2 at 6 dpi) and thin, paste-like feces in C1 (before infection at 0 dpi and between 8 to 10 dpi). 

A marked transient leucocytopenia (<50% to 0 dpi) during the first or second week pi was found in one llama and the pc-goat, but in no other animal (Figure 1G).

### 3.2. Humoral Response to PPRV Is Weak in Camelids but Pronounced in Cattle

In SAC, low antibody levels and a prolonged time until seroconversion (12 to 21 dpi) were detected in all animals with the used competition ELISA (cELISA). Low neutralizing antibody (NAb) titers (1.1–1.8 log10 ND50) were detected in the three alpacas and llama (L) 6, but none in L4 and L5 at the end of the experiment (28 dpi). Of the six experimentally infected dromedaries, two young adult dromedaries (D.6A5, D.DBO) inoculated with a nebulizer seroconverted by showing low antibody levels around the cut-off values of the used cELISA between 20 dpi and the end of the experiment at 74 dpi (Figure 1F). However, the three older dromedaries inoculated with nebulizer (D.No4) or Pasteur pipette (D.F7B, D.No2) and the one young adult dromedary inoculated with Pasteur pipette (D.45A) showed no seroconversion. NAb titers were not analyzed for dromedaries. 

All experimentally inoculated cattle seroconverted at 10 dpi and NAb titers were moderate to high (1.95–2.56 log10 ND50) at the end of the experiment (16 dpi).

No seroconversion was detected in any of the contact-control animals of the three animal trials. 

### 3.3. Molecular Detection of PPRV-RNA in Swab Samples from Camelids and Cattle 

Weak to low PPRV-RNA loads (quantification cycle value [Cq] Cq ≥ 33.85) were detected in oronasal swabs in all three alpacas (A1–3) (3 to 10 dpi; Cq 39.24–33.85) and in one of three llamas (L5) (4 to 10 dpi; Cq 38.37–37.51), and weak loads in one llama fecal swab (L6 at 14 dpi; Cq 38.61) and in one whole-blood sample (Cq 37.68) from one alpaca (A3 at 8 dpi). Conjunctival swabs from all SAC were PCR-negative. No PPRV-RNA was detected in any of the serum, whole-blood or swab samples from L4 and the serum, whole-blood or swab samples from the four tested dromedaries that showed a weak serological antibody response at 20 dpi. Of the pc-goat, blood (3 to 20 dpi) and swab samples (3 until 20 dpi or euthanasia at 27 dpi) were positive by PCR (Figure 1A–E). 

In cattle, PPRV-RNA loads were weak to moderate in oronasal (1–9 dpi; Cq 38.37–28.22), weak to low in conjunctival (5–10 dpi; Cq 41.90–32.23) and weak in fecal swabs (7–8 dpi; Cq 38.28–35.48). Whole-blood and serum samples of C1–3 were all PCR-negative (Figure 1A–E). 

PPRV-RNA excretions ceased in cattle and SAC after seroconversion. No virus was isolated from any of the PCR-positive swab and tissue samples from both cattle and SAC. Similarly, PPRV was not isolated from any swab sample from the six dromedaries and two contact control goats of trial 3, but a few swab samples from the pc-goat were positive by virus isolation (Figure 1A–C). 

### 3.4. PPRV-Antigen or RNA Detection in Lymphoreticular Tissue from SAC and Cattle

#### 3.4.1. Gross Pathological Results

Dromedaries were not euthanized at the end of the experiment. Hence, no tissue samples were available for virological analyses. 

In accordance with the absence of obvious clinical signs in SAC and cattle at the end of the animal trials, no gross pathological alterations or lesions typical for a PPRV infection were detectable at post-mortem examination. 

In SAC, gross examination revealed a tonsillitis in one llama (L4) and a severe (L5) or mild to moderate (L6) follicular hyperplasia of the tonsils in two llamas. One alpaca (A3) showed a conjunctivitis and the contact control llama (L7) showed an ecchymosis of the conjunctiva. 

#### 3.4.2. Molecular, Histopathological, and Immunohistochemical Results

In general, about half of the organ samples of SAC positive by PCR (24.1%, 7/29) were also positive by IHC (12.5%, 3/24) at 28 dpi. In contrast, a considerably higher proportion of organs of the infected cattle were positive by PCR (50.0%, 12/24), while no organ samples were positive by IHC (0.0%, 0/19) at 17 dpi.

##### South American Camelids

In general, weak to low PPRV-RNA loads were detected in tonsil (A1), head (A1 to A3, L6), lung associated lymph nodes (A1, L6), and in jejunal Peyer’s patches (A1). 

**Figure 2:**
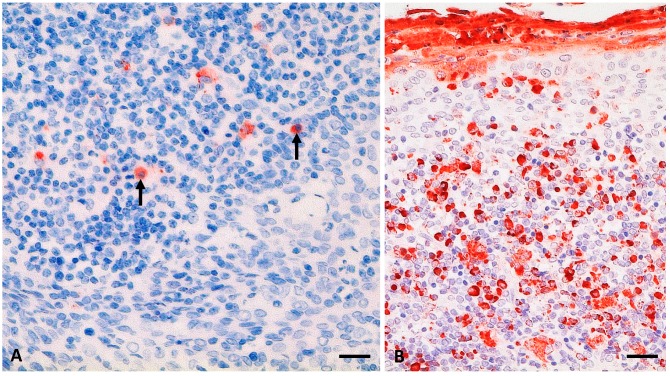
PPRV antigen (-ag) detection (in red) in palatine tonsil from an alpaca (A2) at 28 days post infection (dpi) (**A**; see also Table 2) and a goat (positive control; G1) at 9 dpi (**B**) after experimental infection with the PPRV Kurdistan/2011 strain. (**A**) mild PPRV-ag detection (6% to 25% positive cells) in single lymphoid cells of an enlarged section of a lymphoid follicle of the tonsil from A2, including lymphocytes (arrows); (**B**) positive control, palatine tonsil section from a clinically severely affected goat with severe accumulation of PPRV-ag (>75% positive cells) in mononuclear lymphoid cells as well as epithelia and desquamated material. Immunohistochemistry, monoclonal mouse anti-PPRV-Np (IDvet); scale bar indicates 20 µm.

With IHC, weak to mild staining reactions against PPRV-ag were obtained in the tonsils of two alpacas (A2, A3) (Figure 2, Table 2). Interestingly, tonsils of the alpacas A2 and A3 showed an unspecific mild, acute, necrotizing tonsillitis, which in A2 was associated with an immunohistochemical PPRV-ag detection in crypt epithelium and lymphoreticular tissue. Although PPRV-ag in both alpacas was mainly found sub-epithelially (A2) or in the sinus (A3) in mononuclear cells, a few follicles were also IHC-positive (Figure 2A). 

Mild loads of PPRV-ag were found in parenchymal or lymphoreticular cells in the caecum of A2 and in the colon of A3 (Table 2). In addition, single lymphocytes and plasma cells in the intestinal mucosa (A2, A3) or submucosa (A3) were IHC-positive. In the caecum of A2, a few follicles showed IHC-positive results in lymphocytes of the germinal center. 

An eosinophilic enteritis associated with the detection of parasites (protozoa, nematodes) was found in all SAC (including the contact control L7). 

##### Cattle

Weak to low PPRV-RNA loads were detected by PCR in the lymphoreticular system: PPRV-RNA was detected in the buccal mucosa (C3), follicular area of the third eye lid, palatine tonsils, and retropharyngeal lymph nodes, head and lung associated lymph nodes and in Peyer’s patches of the small intestine (C1, C2, C3) (Table 2). The three cattle showed an eosinophilic enteritis—indicating a subclinical, mild endoparasitosis—as well as an unspecific mild, lymphohistiocytic conjunctivitis and unspecific lesions in nasal and conjunctival tissue. In accordance to the mild to absent clinical signs in cattle, no obvious evidence of a PPRV infection was found by histopathological analyses or IHC. IHC staining consistently revealed negative results for all cattle tissue examined. 

### 3.5. RT-qPCR Is the Most Sensitive Virological Method for Diagnosis of PPRV-Infection in SAC and Cattle

Of the ten cattle and the ten SAC samples of different matrices (swab, tissue, blood) positive for PPRV-RNA by RT-qPCR, the antigen-capture (ag)-ELISA detected only one oronasal swab sample from one animal (cattle C1, 6 dpi; Cq 30.73) as positive (1 of 20), but another oronasal cattle sample with a higher PPRV-RNA load (cattle C3, 5 dpi; Cq 28.22) tested negative with this assay. None of the PCR positive samples were detected positive by LFD and virus isolation (Table 3). 

## 4. Discussion

Cattle are considered dead-end hosts for PPRV [2,18,52]. Furthermore, multiple disease outbreaks and fatalities in camels associated with PPRV-infection with lineages LII, LIII [15], and LIV strains [12,16,17] have suggested PPR is an emerging disease in camels [12,15,16,17]. However, clinical and virological results of field and experimental studies of PPRV-infection in camels and cattle are contradictory [11,14,17,52,53,54]. There is no scientifically sound evidence that cattle or camelids may transmit the disease to other susceptible animals and act as reservoir hosts for PPRV to our knowledge. To elucidate the potential role of cattle and camelids in the epidemiology of PPRV, we herein combine the results of the transmission trials with cattle, SAC and dromedaries with a comprehensive literature review of experimental and field studies of PPRV-infection in cattle and camelids. 

### 4.1. Clinical Signs Associated with PPRV-Infection

We observed no or only transient mild or unspecific clinical signs in PPRV-infected cattle and camelids using the PPRV LIV strain Kurdistan/2011 that was found virulent in wild and domestic goats and sheep and also in pigs and wild boar [3,9,41]. The positive control (pc)-goat showed mild to moderate clinical signs (Figure 1H) as reported previously for goats [4,9]. The eosinophilic enteritis in all cattle and SAC (including the contact control llama L7) indicates an association of the short transient soft feces in one cattle (C1) before (0 dpi) and after infection (8–10 dpi; 7–8 dpi weak PCR-positive; Figure 1C) and one alpaca (A2) at 7 dpi (PCR-negative) with a previous infection with endoparasites in the intestinal tract rather than by PPRV-infection. The transient and mild nasal (PCR-positive between 1 and 9 dpi) and ocular secretions (PCR-positive between 5 and 10 dpi) in cattle (between 3 and 13 dpi) as well as the histopathologically determined conjunctivitis (PCR and IHC-negative swab and tissue samples) in one alpaca (A1) and the contact control llama (Figure 1) could have been caused by repeated swab sampling. Hence, due to histopathological, IHC and PCR results (Table 2, Figure 1), an association of the mild, unspecific clinical signs with PPRV-infection cannot be determined. Furthermore, rectal body temperatures remained physiologically normal in cattle and camelids (Figure 1I), while body temperatures are often increased during the acuteness of PPRV infection in other hosts such as small ruminants and suids [4,9]. 

Mornet, et al. [55] described fatalities in two of six cattle after PPRV-infection with organ suspensions (by unknown inoculation route) obtained from PPRV-infected goats, but the cause of fatality of the two cattle was attributed to their poor general condition, not to PPRV-infection. The other four cattle showed no obvious PPR-like clinical signs [55]. The lack of PPR-like clinical signs in cattle reported by Mornet, et al. [55] is in accordance with our study results and previous reports by Couacy-Hymann, et al. [52] and Sen, et al. [14] about subclinical PPRV-infection in cattle experimentally s.c. infected with PPRV strains from any one of the four lineages [52] and cattle experimentally infected s.c. or by contact with PPRV-infected goats with a LIV strain [14], respectively (Table S2a). In accordance with the published [14,52,55] and our experimental study results of PPRV-infection in cattle, no clinical signs associated with PPRV infection have so far been reported or observed in cattle or buffaloes in the field (Table S2b) to the knowledge of the authors. 

In dromedaries, i.v. or s.c. infection with virulent PPRV field (LIV, LIII) or attenuated PPRV vaccine (LIII) strains did not result in clinical signs [54,56]. Similarly, camels experimentally infected with different RPV strains of varying degrees of virulence by different inoculation routes (s.c., i.v., contact infection by RPV-infected camels) showed no obvious clinical signs but a transient increase in body temperature in some animals as reported by Taylor [21] and Singh and Ata [57] (Table S2a).

In field studies that reported disease outbreaks in camels associated with or without the detection of PPRV-infection, clinical signs described in camels varied considerably, but were generally similar to those reported in domestic and wild small ruminants. Accordingly, clinical signs in camels included sudden death, fever, respiratory distress, nasal discharge, diarrhea, and abortion [5,12,15,16,41,58,59]. On the other hand, the detection of PPRV antibodies in up to 14% of camels as well as the detection of PPRV, PPRV antigen and RNA in lung samples (see Section 4.2. PPRV-detection in camels) without the report of clinical signs in different field studies suggest that camels are susceptible to PPRV-infection but are resistance to PPRV-induced disease [11,53,60]. Clinical signs of PPR disease are generally not specific and differential diagnoses or concurrent diseases such as bluetongue (BT), food and mouth disease (FMD), contagious ecthyma (orf), contagious caprine pleuropneumonia (CCPP), pasteurellosis, heartwater, and coccidiosis have to be considered [18]. 

### 4.2. PPRV Detection in Camels

Confusion about the impact of PPRV-infection on clinical disease expression in camels might have been caused by a sampling bias, differences in the methods used for the detection of PPRV-infection (Table S2) and the possible influence of concurrent infections. A small number of camels were tested and found positive for infectious PPRV, PPRV-RNA, or PPRV-antigen during large PPR-like disease outbreaks in camels [15,16,17,61,62] (Table S2b) that complicate the interpretation of the study results concerning the epidemiological significance. A high mortality rate (up to 70%) was associated with the isolation of PPRV LII (96 CAMEL 1) and LIII (96 CAMEL 2) from two camels and *Streptococcus equi* isolation from two lung samples from camels in Ethiopia [15]. PPRV LIV strains were isolated from 3/3 camel lungs [12] and 1/6 lung or lymph nodes [16] during PPR-like disease outbreaks, while Intisar, et al. [11] isolated unknown strains of PPRV from 5/10 lungs obtained from clinically healthy camels that showed lesions in their lungs upon post-mortem examination at the slaughterhouse [11]. Similarly, a low number of PPRV-RNA or antigen positive samples were reported during large PPR-like disease outbreaks by various authors: PPRV LIV strain in Iran in two camels [17], PPRV-RNA detection of a LIII strain (Kenya_PPRV_Camel_Mandera) in an unknown sample matrix from 1/25 camels with clinical signs in Kenya [61], and antigen or RNA of PPRV LIV strains in 6/6 and 5/6 lungs and lymph nodes, respectively, in camels in Sudan [16]. A higher proportion of camel samples were found positive by Kwiatek, et al. [12] who sequenced PPRV LIV-RNA from 38/49 lung, liver or spleen from 80 field samples from camels with PPR-like clinical signs in Sudan. In slaughterhouse studies, Intisar, et al. [11] found 33.6% of 220 (locally up to 48.5%) lung samples positive for PPRV antigen in clinically healthy camels with lesions in their lungs, and Saeed, et al. [53] detected PPRV antigen in 45.1% (214 of 474) lung samples showing pneumonia from clinically healthy camels. Interestingly, PPRV-antigen detection in 32/214 PPRV-antigen positive lungs was associated with the detection of antigen of one or two other respiratory viruses (bovine virus diarrhea virus, parainfluenza virus 3, respiratory syncytial virus, bovine herpes virus-1, adenovirus) in the study of Saeed, et al. [53] (Table S2b). However, whether these pathogens may also be detected in lung samples without lesions or pneumonia was not evaluated in the two slaughterhouse studies [11,53]. Nevertheless, the field studies of Roger, et al. [15], Intisar, et al. [11] and Saeed, et al. [53] demonstrated that mixed infections with different respiratory pathogens should be considered when evaluating possible causes of respiratory disease expression in PPRV-infected camels. Whether clinical signs may be reproduced in camels by experimental infection using PPRV strains isolated from camels to prove causal relationship according to Koch’s postulates, has never been investigated so far to our knowledge. 

Contradictory reports about the susceptibility and role of camels in RPV epidemiology are also available for RP field studies speculating about potential RP disease in camels during RP outbreaks in cattle despite the lack of diagnostic evidence [57,63]. However, RP disease could not be reproduced in camels experimentally infected with different virulent and attenuated strains of RPV [21,57]. 

Concurrent infections with other pathogens, general condition and nutrition status and breed may also influence the susceptibility of individual hosts of PPRV [4,9,64]. An association of a poor body condition score with a higher PPRV seroprevalence rate (poor 16.67%, fair 3.43%, and good 2.39%) was reported in a field study with 1517 camels in Nigeria [64], and PPRV-RNA (by RT-PCR and sequencing). Furthermore, PPRV-antigen detection was associated with *Streptococcus equi* isolation from lung samples from camels during a large disease outbreak in Ethiopia (see earlier) [15]. Immune suppression evident by severe leukocytopenia is a typical cause of infection with various morbilliviruses including PPRV [4,9,39]. Indeed, in the present study, one llama and the pc-goat showed leukocytopenia (<50% of 0 dpi) (Figure 1G), although we cannot exclude that immune suppression was caused by other factors. 

Differences in the susceptibility to PPRV-infection by species or breed have not been investigated so far for dromedaries and Bactrian camel species or species hybrids. Dromedaries are resistant to FMD and do not contract the disease, while Bactrian camelids show obvious clinical signs and can contract FMD [65], which might also be attributable to PPRV. Unfortunately, many studies lack information about the camel species or breed involved (Table S2). 

### 4.3. PPRV-Transmission

So far, no isolation of infectious PPRV or of PPRV-RNA from se- or excretions from camels or cattle has been documented: No PPRV-RNA could be detected in secretion or excretion of cattle subcutaneously (PPRV LI to IV [52]; PPRV LIV [14]) or dromedaries intravenously (PPRV LIV [54]) infected with PPRV. Also in our study, we could not isolate infectious PPRV from any of the PCR-positive swab and tissue samples from cattle and SAC. Although in cattle the highest PPRV-RNA load in an oronasal swab sample (Cq 28.22) was found below the cut-off value of Cq ≤ 31 that correlated with a successful isolation of PPRV in cell culture for samples from suids and small ruminants experimentally infected and analyzed with the same PPRV strain and PCR assay, respectively [9].

Accordingly, no proof of contact transmission of PPRV from cattle (PPRV LI-IV) [52] or camels (PPRV LIV) [54] experimentally infected with PPRV to susceptible contact goats or other artiodactyls has been described in any sound experimental or field study (Table S2). El-Hakim [19] described subclinical or mild respiratory disease with cough, nasal discharge and fever associated with or without PPRV infection in some camels in Saudi Arabia and the transmission of a PPRV LI strain (of at least three tested PPRV lineages) from camels to goats. The source of PPRV infection in the camels and goats as well as biosafety and sanitary measures applied, if any, to preclude natural or concurrent infections with PPRV or especially other pathogens, remain unclear. 

We found no contact transmission of the virulent PPRV LIV strain from cattle to contact control goats and from SAC and dromedaries to contact control goats and a llama or dromedaries (Figure 1), respectively, confirming the results of Couacy-Hymann, et al. [52] for cattle and of Fakri, et al. [54] for camels. A possible reason for the detection of PPRV-RNA in nasal, conjunctival or fecal swab samples of cattle and SAC in the present study might be attributed to using the more natural intranasal route of infection, while Couacy-Hymann, et al. [52] and Fakri, et al. [54] used s.c. and i.v. infection, respectively. On the other hand, no PPRV-RNA was detected in swab samples collected between 0 and 14 dpi from Indian cattle that were infected by contact to PPRV-infected goats [14], and no PPRV-RNA was detected in swab samples from dromedaries that were intranasally inoculated with PPRV in the present study (Figure 1A–C). 

Therefore, besides the inoculation route and virus dose, the virulence of different virus strains and the susceptibility of a host may influence the pathogenesis in different animal species [26] (see also Section 4.5. Pathogenesis, virus and host factors). Subcutaneous vaccination of camels with a PPRV LIII vaccine [56], infection of cattle with a mild PPRV LII strain [52] and vaccination of camels with an RPV vaccine [57] revealed lower seroconversion rates compared to animals infected by s.c., i.v. or by contact infection using virulent PPRV (LI, LIII, LIV in cattle and LIII and LIV in dromedaries), and RPV strains [21,52,54,56,57]. This indicates that the virulence of a PPRV strain plays a more important role than the inoculation route for at least the humoral immune response. 

For our transmission trials, we used the same virus stock (PPRV LIV Kurdistan/2011), intranasal inoculation route, dose and volume (2 x 10^4.5 TCID50/ml) for experimental infection of both cattle and camelids—which has been proven before to be virulent in small ruminants and suids in previous experiments [3,9]. Nevertheless, in contrast to suids and small ruminants [3,9], PPRV-RNA excretion in cattle and SAC ceased after seroconversion and dromedaries (Figure 1A–C) shedded no PPRV-RNA. The considerable differences in PPRV-RNA loads in oronasal, conjunctival and, in particular, in fecal swab samples from cattle and SAC over time by animal species in comparison to recent data sets from suids and small ruminants [9] were in accordance with results of the statistical analysis using a linear mixed-effects (lme) model and independent 2-group Mann–Whitney test with Bonferroni correction (details in Table S3). Fecal shedding of PPRV-RNA over time was significantly lower by cattle than from other artiodactyls, except from SAC using the lme model. Interestingly, PPRV excretion in fecal samples from suids and small ruminants seem to be a major source of PPRV transmission [9], while the lack or short-time PPRV-RNA excretion in feces from camelids and cattle, respectively, indicate that PPRV-spread by feces is not a likely source of PPRV-infection (Table S3, Figure 1C). 

### 4.4. PPRV Antibody Response and Performance of Diagnostic Methods

In the present study, the time of seroconversion in cattle (10 dpi) and antibody levels as measured by competitive ELISA (cELISA) were comparable with the antibody progression reported for suids and small ruminants [9]. NAb titers in cattle were similarly moderate to high (1.95–2.56 log10 ND50) at 17 dpi than in sheep (1.76–2.56 log10 ND50) at 21 dpi but generally lower than those in suids between 16 and 30 dpi and goats at 11 to 15 dpi (2.16–2.96 log10 ND50) [9]. 

In SAC, low antibody levels and a prolonged time until seroconversion were detected in all animals using the cELISA (seroconversion between 12 and 21 dpi). Low NAb titers (1.1–1.8 log10 ND50) were detected in the three alpacas and L6 but no NAbs at all in L4 and L5 at 28 dpi. Of the six experimentally infected dromedaries, two young animals (D.6A5, D.DBO) seroconverted with antibody levels around the cut-off of the cELISA at 20 dpi. However, none of the other four dromedaries seroconverted showed a clear increase in antibody levels. Similarly, Fakri, et al. [54] found a prolonged seroconversion between 14 and 18 dpi in five dromedaries experimentally infected with a virulent LIV strain and low to moderate (1.02–2.46 log10 ND50) NAbs after 35 dpi.

Intisar, et al. [11] reported a considerably lower seroprevalence in a field study of PPRV infection in Sudanese camels of 2.1% (41 of 1988) despite a considerably higher proportion of camel lungs detected positive for PPRV-antigen (33.6% of 220). Differences in populations of immunoglobuline G (IgG) isotypes including the additional heavy-chain antibodies found in camel blood but not in other mammals was suggested as a possible reason for the lower seroprevalence by Intisar, et al. [11]. Indeed, lower antibody levels were found in SAC compared to domestic ruminants infected with BTV, SBV, or WNV, respectively [11,66,67]. 

The comparison of virological test systems with swab, tissue or blood samples from cattle and SAC revealed that a validated RT-qPCR targeting the N segment of PPRV is the most sensitive method for the detection of a recent PPRV-infection in these animals until seroconversion (Table 3). In contrast, the used PPRV-antigen ELISA targeting the N protein, the LFD targeting the H gene of PPRV and endpoint dilution assays for analysis of virus titers in animal serum samples were not found suitable diagnostic methods for cattle and SAC. One likely reason is the transcription gradient resulting in higher transcription rates of the N compared to the H gene [32]. 

### 4.5. Pathogenesis, Virus, and Host Factors

PCR-results obtained from samples taken during gross examination confirmed that a combination of different samples collected from head and lung associated lymph nodes, palatine tonsils, mesenterial lymph nodes and small intestinal Peyer’s patches is also most suitable for post-mortem PPRV-diagnosis in cattle and SAC (Table 2) as suggested for suids and small ruminants [4,9]. Albeit PPRV-RNA loads were low or not detectable in the examined tissue from cattle and SAC, the generally similar tissue distribution of PPRV-RNA in tissue of cattle and camelids compared with small ruminants and suids contrast the considerable variations particularly of virological results between cattle and camelids with the other artiodactyl species, including the lack of viremia (see earlier). 

Interestingly, the pathogenesis found in cattle and camelids (particularly in SAC) in the present study resembles the pathogenesis in ferrets experimentally infected with SLAM-blind CDV [37]. None of the ferrets transmitted the virus to CDV-naïve contact-control ferrets, they presented no clinical signs and no viremia, except for one ferret that showed a transient mild viremia probably due to three compensatory mutations in the H gene of the SLAM-blind virus [37]. Nevertheless, all ferrets elicited a solid humoral immune response that was attributed to low-level replication of CDV in nectin-4 expressing epithelial cells [37]. Cattle experimentally infected with PPRV also developed a rapid and solid humoral immune response, while camelids developed a prolonged and lower or no antibody response possibly due to species-specific differences in the immune response (see section humoral immune response) or a lower susceptibility, particularly of dromedaries, to PPRV-infection than cattle. Whether morbilliviruses initially infect immune cells via the SLAM-receptor and/or initially infect epithelial cells via the nectin-4 receptor has been controversially discussed [4]. Nevertheless, the results of the ferret trial with SLAM-blind CDV suggest that local replication in the respiratory epithelium may facilitate low-efficiency transmission [37]. Consequently, if PPRV infection of dead-end hosts resembles infection of typical hosts with a SLAM-blind virus, virus shedding and transmission cannot totally be excluded for dead-end hosts of PPRV, for example cattle and camelids, but is not very likely. By definition, a dead-end host is a host in which a pathogen may cause disease, but not maintain transmission, thus, the pathogen is not efficiently transmitted to other hosts [27,68,69]. 

In typical/maintenance hosts, morbillivirus-infection of (i) SLAM-expressing immune cells (in particular DC, activated lymphocytes, and macrophages) leads to systemic virus dissemination via blood and lymph to other lymphoid organs and (ii) infection of epithelial cells via nectin-4 receptor results in virus shedding without the need of additional exit receptors [4,33,37]. Morbillivirus-infection of immune cells may also lead to their destruction and a consequent immune suppression [4,39]. Leukocytopenia and immune suppression in animals or humans is a typical consequence of infection with different morbilliviruses, such as PPRV, CDV and measles virus (MeV, species: *measles morbillivirus*), respectively [4,9,39,70]. Whether the severe leucocytopenia in one llama and the pc-goat was due to PPRV-infection, remains unknown (Figure 1G). Since cattle and SAC showed a similar pathogenesis for PPRV-infection than ferrets infected with a SLAM-blind virus, infection of epithelial cells in the respiratory tract via nectin-4 appears reasonable. The SLAM receptor gene is less conserved than the nectin-4 receptor gene among species [35], further supporting the hypothesis that the H glycoprotein of PPRV is not (efficiently) binding to the cattle and camelid SLAM-receptors. 

PPRV-RNA detection in more distantly located lymphoid organs of cattle and SAC, such as small intestinal Peyer’s patches (Table 2), suggest that other host cell receptors are involved in PPRV uptake and dissemination—e.g., the C-type lectin dendritic cell-specific intercellular adhesion molecule-3-grabbing non-integrin (DC-SIGN) receptor expressed on DC [71]. A dual role of DC was described for MeV infection since MeV may infect or is captured by DC via DC-SIGN or SLAM receptor [71]. Pope, et al. [4] suggested that tonsils and lymph nodes draining the site of PPRV-inoculation are initial sites of PPRV replication. Indeed, we found PPRV-RNA or antigen in head and lung associated lymph nodes and palatine tonsils (*Tonsilla veli palatini* in suids) in all animal species (goat, sheep, pig, wild boar [9], cattle, and SAC) (Table 2, Figure 2), although post-mortem examination was conducted during or after the peak of PPRV-infection (16 to 30 dpi). The IHC comparison of the palatine tonsil sections from alpaca A2 and the positive control goat (Figure 2) revealed a considerably reduced proportion of PPRV-ag in the alpaca tonsil (mild versus severe). However, the alpacas were euthanized late after experimental infection (28 dpi) in comparison to the goat (9 dpi). A mild proportion of PPRV-ag was detected in the palatine tonsil of a goat during the reconvalescence phase (15 dpi with Kurdistan/2011 strain) after showing moderate clinical signs [9]. In goats intranasally infected with a PPRV LIII strain (CI/89), clinical signs were mild, but moderate to marked proportions of PPRV-ag were detected at 7 dpi or 9 dpi in the follicle/mantel zone, germinal center, diffuse lymphoid tissue, and crypt epithelium of the palatine tonsil. Hence, PPRV-ag may be detected in similar tissue than in goats, but PPRV-ag distribution in alpaca tonsils in the early stage after infection remains to be investigated. 

### 4.6. Possible Impact of Ecology on PPRV Infection and Spread

For multi-host pathogens, pathogen dynamics, persistence (*R*_0_ > 1) or extinction (*R*_0_ < 1) in an ecosystem are determined by the community composition as well as the movement and behavior of hosts by space and time that affect intra- or inter-species contact and therefore pathogen transmission [26,27]. 

Dromedaries kept separately from PPRV-infected domestic ruminants showed no seroconversion to PPRV [59,72], while a considerable proportion of camels, cattle, and buffalo can be found seropositive where reared together with small ruminants (one epidemiological unit) [73]. No seropositive cattle was found in Turkey despite PPRV-infection detected in local small ruminant herds [74], which was attributed to a lack of infection and circulation of PPRV in cattle [74]. In field studies, seroprevalence rates were generally similar or lower in camels and cattle compared to small ruminants. This indicates spillover from PPRV-infected small ruminants to cattle and camels by contact-infection and may explain the varying seroprevalence rates in different regions (see details in Table S2b). The turnover rate of cattle (10%) and likely also of camels is considerably lower than in small ruminants (30%) [33,75,76], indicating that the overall PPRV-infection rate and, therefore, susceptibility to PPRV-infection might be even lower in cattle and camels compared to small ruminants than suggested by the relative seroprevalence rates detected in field surveys. 

## 5. Conclusions

The transmission trials revealed (i) absent or low PPRV-RNA loads in swab samples until seroconversion, (ii) no shedding of infectious PPRV, (iii) lack of PPRV transmission, and (iv) generally subclinical PPRV-infection in experimentally PPRV-infected cattle, SAC and dromedaries. Together with the results of the literature review, we suggest that cattle and camelids are dead-end hosts that generally do not contribute to the spread of PPRV, possibly due to species-specific physiological constraints such as differences in the major natural host cell receptors SLAM on immune cells that considerably impair the infection of cattle or camelid by PPRV. However, additional studies would be helpful to further investigate the course of disease and the capability of PPRV-transmission by cattle and camelids influenced by critical factors such as virus-host interaction, breed, stress (e.g., long desert journeys) or concurrent infections and to finally prove the impact of dead-end hosts. 

In addition, of all compared methods, RT-qPCR and cELISA were found most reliable to confirm PPRV-infection in cattle and camelids before and after seroconversion, respectively. An antigen-ELISA and a pen-side test (lateral flow device, LFD) that allow non-invasive sampling and PPRV-detection in the field were not suitable for PPRV-diagnosis in cattle and camelids (Table 3)—probably due to the low PPRV-RNA loads in their excretions (Figure 1A–E). 

## Figures and Tables

**Table 1:** Overview of animals, study design and outcome of peste-des-petits-ruminants virus (PPRV) transmission trials with cattle, alpacas, llamas, dromedaries and goats using PPRV lineage IV strain Kurdistan/2011 for intranasal infection. Contact control animals were added 2 days (trial 1) or 3 days (trials 2 and 3) after experimental infection (dpi). Seroconversion was detected in all experimentally infected cattle, alpacas and llamas and in 2/6 dromedaries, while PPRV-RNA was detected in 3/3 cattle, 3/3 alpacas and 2/3 llamas but not in any of the PPRV-infected six dromedaries. None of the cattle and camelids excreted infectious PPRV or transmitted PPRV to any of the contact animals.

Trial No.	Trial ID	Intranasally Inoculated Animals			Contact Control Animals			Outcome of Experiment			
		Species * and ID	Sex	Age (Months)	Species * and ID	Sex	Age (Months)	Seroconver-sion (Total No. by Spp.)	Shedding of PPRV-RNA (Total No. by Spp.)	Shedding of Infectious PPRV	Contact Transmission
1	C–G	C1–C3	3 f	3	G5, G6	2 m	4	3 C	3 C	no	no
2	SAC-GL	A1–A3 L4–L6	3 m 3 m	6–10 7–8	L7	1 m	9	3 A, 3 L	3 A, 2 L	no	no
					G8, G9	2 m	12–13	0 G	0 G	no	no
3	D-GD (pen 1)	D.54A, D.6A5, D.BO	3 m	young adult (72)	D.204, D.05E	2 f	young adult (72)	2 D	0 D	no	no
					G.9499, G.9500	2 m	24	0 G	0 G	no	no
3	D-GD (pen 2)	D.54A, D.F7B, D.No2	1 m, 2 f	old (216)	D.No1, D.No6	1 m, 1 f	old (170)	0 D	0 D	no	no
3	D-GD (pen 3)	G.8682 ^†^	1 m	24	none			1 G	1 G	yes	NA

* Scientific names: C, cattle (*Bos taurus taurus*, breed: Holstein Friesian; A, alpaca (*Vicugna pacos*); L, llama (*Llama glama*); SAC, South American camelids; D, dromedary (*Camelus dromedarius*); G, goat (*Capra aegagrus hircus*; breeds: White German goat; German: ‘Weiße Deutsche Edelziege’ (trials 1 and 2), local goat breed of United Arab Emirates [trial 3]); pen, animals were kept in 3 different enclosures—pen 1 to 3; f, female; m, male; ^†^ positive control goat; NA, not applicable since the positive control goat was kept alone in a separate stable.

**Table 2:** PPRV-RNA and antigen detection in selected tissue samples collected from different animal species experimentally intranasally infected with PPRV lineage IV Kurdistan/2011. (**A**) PPRV-RNA detected in tissue samples with real-time quantitative reverse transcription-PCR (RT-qPCR) assay of Batten et al. 2011 [50]. Tissue most suitable for PPRV diagnosis by PCR in the examined species are highlighted in bold. All examined tissue samples from the positive control goat and various tissue samples from three cattle, three alpacas and one llama were PCR-positive. (**B**) PPRV-antigen detected in tissue samples by immunohistochemistry (IHC) using Mab anti-PPRV-Np (purified monoclonal mouse antibody against PPRV nucleoprotein, ID.vet). A few tissue samples from 2/3 alpacas were positive by IHC, while no PPRV-antigen was detected in any of the cattle and llama tissues. A PPRV-negative control goat and the contact control llama (L7 not PPRV-infected by contact) were PCR and IHC negative.

**(A)**												
		**Animal Trial ID**	**C-G**	**C-G**	**C-G**	**SAC-GL**	**SAC-GL**	**SAC-GL**	**SAC-GL**	**SAC-GL**	**SAC-GL**	**D-GD**
		**Animal ID**	**C1**	**C2**	**C3**	**A1**	**A2**	**A3**	**L4**	**L5**	**L6**	**pc-G**
		**dpi**	**17**	**17**	**17**	**28**	**28**	**28**	**28**	**29**	**29**	**35**
**Organ Location**	**Organ ID**	**Tissue (Cq)**										
head	1	third eye lid	33.63	34.11	33.17	-	-	-	-	-	-	39.66
	2	lacrimal gland	-	-	-	-	-	-	-	-	-	34.39
	6	tongue (apex)	nd	nd	nd	-	-	-	-	-	-	nd
	7	**palatine tonsil**	33.26	34.80	30.55	33.88	-	-	-	-	-	32.78
	8	**retropharyngeal ln.**	32.83	32.20	31.45	-	35.32	37.42	-	-	-	31.48
	9	**mandibular ln.**	nd	nd	nd	33.21	34.56	-	-	-	33.28	nd
	10	**parotideal ln.**	nd	nd	nd	35.66	-	-	-	-	-	nd
cervical ^†^	11	trachea	-	-	-	-	-	-	-	-	-	nd
	12	esophagus	-	-	-	-	-	-	-	-	-	nd
thoracal	13	lung	-	-	-	-	-	-	-	-	-	nd
	14	**bronchial ln.**	-	34.84	35.41	34.94	-	-	-	-	32.93	32.28
	15	**mediastinal ln.**	36.07	-	-	32.56	-	-	-	-	35.08	31.07
abdominal	19	**jejunal PP**	36.20	37.52	33.08	33.05	-	-	-	-	-	nd
	20	**ileal PP**	-	38.43	33.82	-	-	-	-	-	-	nd
	21	caecum	-	-	-	-	-	-	-	-	-	nd
	22	colon	-	32.75	-	-	-	-	-	-	-	34.41
	23	**mesenteric ln.**	-	34.69	32.80	-	-	-	-	-	-	32.17
	24	rectum	-	38.43	33.82	-	-	-	-	-	-	nd
	27	spleen	37.86	-	-	-	-	-	-	-	-	34.28
cerebral	30	different brain tissues ^‡^	nd	nd	nd	-	-	-	-	-	-	-
**(B)**												
		**Animal Trial ID**	**C-G**	**C-G**	**C-G**	**SAC-GL**	**SAC-GL**	**SAC-GL**	**SAC-GL**	**SAC-GL**	**SAC-GL**	**D-GD**
		**Animal ID**	**C1**	**C2**	**C3**	**A1**	**A2**	**A3**	**L4**	**L5**	**L6**	**pc-G**
		**dpi**	**17**	**17**	**17**	**28**	**28**	**28**	**28**	**29**	**29**	**35**
**Organ Location**	**Organ ID**	**Tissue (IHC)**										
head	1	third eye lid *	nd	-	-	-	-	-	-	-	-	nd
	2	lacrimal gland	-	-	-	-	-	-	-	-	-	nd
	6	tongue (apex)	nd	nd	nd	nd	nd	nd	nd	nd	nd	nd
	7	palatine tonsil *	-	-	-	-	++	+	-	-	-	nd
	8	**retropharyngeal ln.**	-	-	-	-	-	-	-	-	-	nd
	9	**mandibular ln.**	nd	nd	nd	-	nd	-	-	-	-	nd
	10	**parotideal ln.**	nd	nd	nd	-	-	-	-	-	-	nd
cervical ^†^	11	trachea	-	-	-	-	-	-	-	-	-	nd
	12	esophagus	-	-	-	-	-	-	-	-	-	nd
thoracal	13	lung	-	-	-	-	-	-	-	-	-	nd
	14	**bronchial ln.**	-	-	-	-	-	-	-	-	-	nd
	15	**mediastinal ln.**	nd	-	-	-	nd	nd	-	-	-	nd
abdominal	19	**jejunal PP**	-	-	-	-	nd	-	-	-	-	nd
	20	**ileal PP**	nd	-	-	-	-	-	-	-	-	nd
	21	caecum *	-	-	-	-	+	-	-	-	-	nd
	22	colon *	-	-	-	-	-	+	-	-	-	nd
	23	**mesenteric ln.**	-	-	-	-	-	-	-	-	-	nd
	24	rectum *	-	-	-	-	nd	-	-	-	-	nd
	27	spleen	-	-	-	-	-	-	-	-	-	nd
cerebral	30	different brain tissues ^‡^	nd	nd	nd	nd	nd	nd	nd	nd	nd	nd

Cq, quantitative cycle value; dpi, days after infection; G, goat; IHC, Immunohistochemistry; ln., lymph node; C, cattle; A, alpaca; L, llama; SAC, South American camelids; pc-G, positive control goat; D, dromedary; PP, Peyer’s patches; * results by IHC in lymph reticular system and parenchymal tissue are summarized (the higher positive results are presented); ^‡^ examined brain tissues included choroid plexus, olfactory nerve, optic nerve, optic chiasm, pons, white cerebrum, fourth ventricle (roof), spinal cord (thoracical), medulla oblongata, trigeminal ganglion, as previously described (Schulz et al. 2018 [9]), from the pc-goat only cerebrum was analyzed; ++++, Cq 18–24.999, high PPRV-RNA load/IHC severe; +++, Cq 25–29.999, moderate PPRV-RNA load/IHC moderate; ++, Cq 30–34.999, low PPRV-RNA load/IHC mild; +, Cq 35–42, weak PPRV-RNA load/IHC weak; -, no Cq/IHC negative; nd, not done.

**Table 3:** Results of the comparison of different methods for virological peste-des-petits-ruminants virus (PPRV) diagnosis in cattle and South American camelids (SAC) after experimental intranasal infection with PPRV lineage IV strain Kurdistan/2011. Different sample matrices (swab, tissue, blood) were analyzed from cattle (C), alpacas (A) and llamas (L) using two SLAM-expressing cell lines (VDS and CHS-20) for virus isolation, three PCR assays for real-time quantitative reverse-transcription PCR (RT-qPCR), antigen ELISA (Ag-ELISA) and lateral flow device (LFD). RT-qPCR was found the only suitable virological method for the detection of PPRV infection in cattle and SAC. Similarly, RT-qPCR was previously found most suitable for the detection of PPRV infection in sheep, pigs and wild boar but not LFD (Schulz et al. 2018 [9]). In contrast, for sheep and suids, PPRV isolation with cell culture and antigen detection with Ag-ELISA was possible for selected samples, and detection of PPRV infection was generally possible with all four methods in goats (Schulz et al. 2018 [9]). Samples detected positive are highlighted in bold.

Serial No.	Animal Trial ID	Animal Species	Sample Material		Animal ID	dpi	SLAM-Cells (Max. TCID50/mL on VDS or CHS) *	Detection of PPRV-Np by RT-qPCR (Cq Value)		Ag-ELISA (OD NC %)	LFD (pos/neg)
	(Trial No.)							Bao et al. 2008	Batten et al. 2011		
35	C-G (1)	cattle	oronasal	swab	C3	5	neg	**27.02**	**28.22**	13	neg
36	C-G (1)	cattle	oronasal	swab	C1	6	neg	**32.01**	**30.73**	**46**	neg
37	C-G (1)	cattle	fecal	swab	C3	7	neg	**40.28**	**35.48**	−12	neg
38	C-G (1)	cattle	oronasal	swab	C3	7	neg	**30.16**	**31.43**	15	neg
39	C-G (1)	cattle	conjunctival	swab	C2	7	neg	**32.17**	**32.23**	1	neg
40	C-G (1)	cattle	mediastinal ln.	tissue	C1	17	neg	**44.16**	**36.07**	−12	neg
41	C-G (1)	cattle	bronchial ln.	tissue	C2	17	neg	**36.11**	**34.84**	−17	neg
42	C-G (1)	cattle	palatine tonsil	tissue	C3	17	neg	**30.06**	**30.55**	1	neg
43	C-G (1)	cattle	retropharyng. ln.	tissue	C3	17	neg	**31.84**	**31.45**	−6	neg
44	C-G (1)	cattle	ileal peyer’s patches	tissue	C3	17	neg	**33.36**	**34.11**	−11	neg
45	SAC-GL (2)	llama	oronasal	swab	L5	4	neg	**38.37**	**39.00**	−10	neg
46	SAC-GL (2)	alpaca	oronasal	swab	A1	7	neg	No Cq	**36.58**	15	neg
47	SAC-GL (2)	alpaca	oronasal	swab	A2	8	neg	**39.21**	**33.85**	−13	neg
48	SAC-GL (2)	alpaca	EDTA-blood	blood	A3	8	neg	No Cq	**37.68**	0	neg
49	SAC-GL (2)	llama	fecal	swab	L6	14	neg	No Cq	**38.61**	−24	neg
50	SAC-GL (2)	alpaca	palatine tonsil	tissue	A1	28	neg	No Cq	**33.88**	−21	neg
51	SAC-GL (2)	alpaca	mediastinal ln.	tissue	A1	28	neg	No Cq	**32.56**	9	neg
52	SAC-GL (2)	alpaca	retropharyng. ln.	tissue	A2	28	neg	No Cq	**35.32**	9	neg
53	SAC-GL (2)	llama	mandibular ln.	tissue	L6	29	neg	No Cq	**33.28**	−20	neg
54	SAC-GL (2)	llama	bronchial ln.	tissue	L6	29	neg	No Cq	**32.93**	−20	neg
	PPRV cell culture virus, strain Kurdistan/2011			pos control			**10^5.5**	**18.56**	ND	ND	**pos**

dpi, days after experimental infection; in, infected by intranasal inoculation; ND, not defined; neg, negative; No Cq, Cq = 45; PPRV-Np, PPRV-nucleoprotein gene; pos, positive; RT-qPCR, real-time quantitative reverse transcription-PCR; SLAM cells, cells expressing signaling lymphocyte activation molecule (CD150); VDS, ‘Vero.dog.SLAM.tag’ vero cells expressing the dog SLAM protein (von Messling et al. 2003 [43]); CHS-20, Monkey CV1 cell line expressing the goat SLAM protein (Adombi et al. 2011 [42]); LFD, lateral flow device, PESTE-TEST, Field test for Peste des Petits Ruminants Virus Infection, BDSL IRVINE LIMITED and The Pirbright Institute, Pirbright, UK, detecting PPRV H protein; Ag-ELISA, ID Screen® PPR Antigen Capture sandwich ELISA, ID.vet, detecting PPRV N protein; OD NC %, optical density % negative control (neg < 20%; pos ≥ 20%); * virus titration was conducted on VDS and CHS cells after freezing and thawing (note: for swab samples titers may be up to 10^2.5 TCID50/ml higher after freezing-thawing (Schulz et al. 2018 [9]).

## Supplementary Materials

The following are available online at https://www.mdpi.com/1999-4915/11/12/1133/s1, Table S1a: Clinical score sheet for the evaluation of clinical signs in PPRV-infected cattle, Table S1b: Clinical score sheet for the evaluation of clinical signs in PPRV-infected South American camelids and dromedaries, Table S2a: Literature overview of animal trials with cattle, buffaloes and camels experimentally infected with peste des petits ruminants virus and with camels experimentally infected with rinderpest virus (RPV), Table S2b: Literature overview of field studies with cattle, buffaloes and camels naturally infected with peste des petits ruminants virus and with camels naturally infected with rinderpest virus, Table S3: Statistical results of Cq values obtained from swab samples collected from different Artiodactyla species over time during transmission trials.
