# Supplementary material for: Camelids and Cattle Are Dead-End Hosts for Peste-des-Petits-Ruminants Virus
Source: Viruses. 2019 Dec 8;11(12):1133. doi: 10.3390/v11121133 (PMC6950723; doi:10.3390/v11121133)
Supplement: Supplementary file 1 [file viruses-11-01133-s001.zip › PCR data_PPRV cattle_trial 1.pdf]

Real-time quantitative reverse transcription-PCR results (Cq values) of cattle (C) after experimental intranasal infection with PPRV lineage IV Kurdistan/2011 strain obtained during cattle-goat transmission trial 1 using PPRV-PCR assay of Batten et al. 2011.

oronasal swabs

days post infection	0	1	2	3	4	5	6	7	8	9	10	11	12	13	14	16
C1	45	45	36.43	37	31.06	38.07	30.73	35.35	34.51	45	45	45	45	45	45	45
C2	45	45	45	45	34.72	38.07	45	35.68	36.74	37.72	45	45	45	45	45	45
C3	45	37.53	29.46	34.54	45	28.22	37.09	31.43	33.45	45	45	45	45	45	45	45

conjunctival swabs

days post infection	0	1	2	3	4	5	6	7	8	9	10	11	12	13	14	16
C1	45	45	45	45	45	38.25	35.67	35.24	36.93	36.16	38.13	45	45	45	45	45
C2	45	45	45	45	45	45	32.49	32.23	36.16	36.57	45	45	45	45	45	45
C3	45	45	45	45	45	38.55	35.93	35.16	34.6	45	45	45	45	45	45	45

fecal swabs

days post infection	0	1	2	3	4	5	6	7	8	9	10	11	12	13	14	16
C1	45	45	45	45	45	45	45	38.28	38.09	45	45	45	45	45	45	45
C2	45	45	45	45	45	45	45	38.08	45	45	45	45	45	45	45	45
C3	45	45	45	45	45	45	45	35.48	35.58	45	45	45	45	45	45	45

whole blood

days post infection	0	1	2	3	4	5	6	7	8	9	10	11	12	13	14	16
C1	45		45		45		45		45		45		45		45	45
C2	45		45		45		45		45		45		45		45	45
C3	45		45		45		45		45		45		45		45	45

serum

days post infection	0	1	2	3	4	5	6	7	8	9	10	11	12	13	14	16
C1	45		45		45		45		45		45		45		45	45
C2	45		45		45		45		45		45		45		45	45
C3	45		45		45		45		45		45		45		45	45
