# Supplementary material for: Camelids and Cattle Are Dead-End Hosts for Peste-des-Petits-Ruminants Virus
Source: Viruses. 2019 Dec 8;11(12):1133. doi: 10.3390/v11121133 (PMC6950723; doi:10.3390/v11121133)
Supplement: Supplementary file 1 [file viruses-11-01133-s001.zip › PCR data_PPRV SAC_trial 2.pdf]

Real-time quantitative reverse transcription-PCR results (Cq values) of alpacas (A) and llamas (L) after experimental intranasal infection with PPRV lineage IV Kurdistan/2011 strain obtained during South American camelid (SAC)-goat and llama transmission trial 2 using PPRV-PCR assay of Batten et al. 2011.

oronasal swabs

days post infection	0	3	4	5	6	7	8	9	10	12	14	17	21	25	28
A1	45	39.14	39.35	38.93	45	36.58	45	45	37.55	45	45	45	N/A	45	45
A2	45	45	45	45	38.13	45	33.85	45	45	45	45	45	N/A	45	45
A3	45	45	37.36	45	38.2	45	45	45	45	45	45	45	N/A	45	45
L4	45	45	45	45	45	45	45	45	45	45	45	45	N/A	45	45
L5	45	45	38.37	37.69	38.3	45	45	45	37.51	45	45	45	N/A	45	45
L6	45	45	45	45	45	45	45	45	45	45	45	45	N/A	45	45

conjunctival swabs

days post infection	0	3	4	5	6	7	8	9	10	12	14	17	21	25	28
A1	45	45	45	45	45	45	45	45	45	45	45	45	N/A	45	45
A2	45	45	45	45	45	45	45	45	45	45	45	45	N/A	45	45
A3	45	45	45	45	45	45	45	45	45	45	45	45	N/A	45	45
L4	45	45	45	45	45	45	45	45	45	45	45	45	N/A	45	45
L5	45	45	45	45	45	45	45	45	45	45	45	45	N/A	45	45
L6	45	45	45	45	45	45	45	45	45	45	45	45	N/A	45	45

fecal swabs

days post infection	0	3	4	5	6	7	8	9	10	12	14	17	21	25	28
A1	45	45	45	45	45	45	45	45	45	45	45	45	N/A	45	45
A2	45	45	45	45	45	45	45	45	45	45	45	45	N/A	45	45
A3	45	45	45	45	45	45	45	45	45	45	45	45	N/A	45	45
L4	45	45	45	45	45	45	45	45	45	45	45	45	N/A	45	45
L5	45	45	45	45	45	45	45	45	45	45	45	45	N/A	45	45
L6	45	45	45	45	45	45	45	45	45	45	38.61	45	N/A	45	45

whole blood

days post infection	0	3	4	5	6	7	8	9	10	12	14	17	21	24	28
A1	45	N/A	45	N/A	45	N/A	45	N/A	45	45	45	45	45	45	45
A2	45	N/A	45	N/A	45	N/A	45	N/A	45	45	45	45	45	45	45
A3	45	N/A	45	N/A	45	N/A	37.68	N/A	45	45	45	45	45	45	45
L4	45	N/A	45	N/A	45	N/A	45	N/A	45	45	45	45	45	45	45
L5	45	N/A	45	N/A	45	N/A	45	N/A	45	45	45	45	45	45	45
L6	45	N/A	45	N/A	45	N/A	45	N/A	45	45	45	45	45	45	45

serum

days post infection	0	3	4	5	6	7	8	9	10	12	14	17	21	24	28
A1	45	N/A	45	N/A	45	N/A	45	N/A	45	45	45	45	45	45	45
A2	45	N/A	45	N/A	45	N/A	45	N/A	45	45	45	45	45	45	45
A3	45	N/A	45	N/A	45	N/A	45	N/A	45	45	45	45	45	45	45
L4	45	N/A	45	N/A	45	N/A	45	N/A	45	45	45	45	45	45	45
L5	45	N/A	45	N/A	45	N/A	45	N/A	45	45	45	45	45	45	45
L6	45	N/A	45	N/A	45	N/A	45	N/A	45	45	45	45	45	45	45

N/A, not analysed
