# Supplementary material for: Camelids and Cattle Are Dead-End Hosts for Peste-des-Petits-Ruminants Virus
Source: Viruses. 2019 Dec 8;11(12):1133. doi: 10.3390/v11121133 (PMC6950723; doi:10.3390/v11121133)
Supplement: Supplementary file 1 [file viruses-11-01133-s001.zip › viruses-624316-supplementary.pdf]

Table S1a. Clinical score (CS) sheet for the evaluation of clinical signs in PPRV-infected cattle.
Table adapted for cattle according to Stober [1] using the clinical score sheet for PPRV-infected small ruminants proposed by Pope et al. [2].

Clinical Score	General signs	Rectal body temperature (°C)	Ocular/nasal discharge	Facial mucosal lesions	Faeces	Respiratory symptoms
**0**	Normal (alert, curious, promptly stands up)	≤39.5	None	None	Normal (*formed*)	Normal respiration rate (*calve: 30-45/min*)
**1**	Mildly inactive (somewhat tired)	>39.5 but ≤40	Watery ocular discharge	Congested oronasal mucosa and buccal papillae	Mild diarrhoea	Mild tachypnoea
**2**	Mildly inactive and depressed, mild inappetance	>40 but ≤41	Watery to mucoid oculonasal discharge: reddened eyes and mild conjunctivitis	Pin-prick lesions within buccal cavity, with some becoming more extensive	Runny	Tachypnoea/ mild cough
**3**	inactive, apathetic, restless and anorexic	>41 or<br>>39.5 for >5 days	Mucopurulent nasal discharge and/or severe conjunctivitis with mucopurulent ocular discharge	Clear erosive lesions on oronasal mucosae; severely congested/oedematous buccal papillae	Frank diarrhoea	Tachypnoea and dyspnoea/ coughing
**4**	Severe depression, unable to stand, extreme lethargy, dehydration	>41 or<br>>39.5 for >5 days followed by rapid fall of temperature (<38.5)	Mucopurulent nasal discharge and severe conjunctivitis with profuse mucopurulent ocular discharge	Severe erosive/ulcerative lesions throughout buccal cavity, nasal mucosa and nares; oedematous lips and erosions on vulval labia	Muco-haemorrhagic diarrhoea	Marked tachypnoea / dyspnoea/ cough

End point definition: When animal reaches a score of 20 they need to be killed on ethical grounds. The decision to euthanasia would additionally be based in the following criteria: 1) A score of 4 is achieved in „General Signs“; 2) A score of 3 is achieved in „General Signs“ for 2 complete, consecutive days and score of 10 or greater is achieved in other categories; 3) A score of 2 is achieved in „General Signs“ for 2 complete, consecutive days and score of 15 or greater is achieved in other categories. CS criteria in italic were modified from the CS sheet published by Pope et al. [2] according to Stober [1].

Table S1b. Clinical score (CS) sheet for the evaluation of clinical signs in PPRV-infected South American camelids (SAC) and dromedaries (D). Table adapted for camelids according to Fowler [3] using the clinical score sheet for PPRV-infected small ruminants proposed by Pope et al. [2].

Clinical Score	General signs	Rectal temperature* (°C) SAC / D	Ocular/nasal discharge	Facial mucosal lesions	Faeces	Respiratory symptoms
**0**	Normal (alert, curious, promptly stands up)	*≤38.9 / ≤38.0*	None	None	Normal (*formed*)	Normal respiration rate (*10-30/min*)
**1**	Mildly inactive (somewhat tired)	*>38.9 but ≤39.5 / >38.0 but ≤38.5*	Watery ocular discharge	Congested oronasal mucosa and buccal papillae	Mild diarrhoea (*soft*)	Mild tachypnoea
**2**	Mildly inactive and depressed, mild inappetance	*>39.5 but ≤40.5 / >38.5 but ≤40.0*	Watery to mucoid oculonasal discharge: reddened eyes and mild conjunctivitis	Pin-prick lesions within buccal cavity, with some becoming more extensive	Runny	Tachypnoea/ mild cough
**3**	inactive, apathetic, restless and anorexic	*>40.5 / 40.0 or >38.9 / 38.0* for >5 days	Mucopurulent nasal discharge and/or severe conjunctivitis with mucopurulent ocular discharge	Clear erosive lesions on oronasal mucosae; severely congested/oedematous buccal papillae	Frank diarrhoea	Tachypnoea and dyspnoea/ coughing
**4**	Severe depression, unable to stand, extreme lethargy, dehydration	*>40.5 / 40.0 or >38.9 / 38.0* for >5 days followed by rapid fall of temperature *(<37.5 / <36.0)*	Mucopurulent nasal discharge and severe conjunctivitis with profuse mucopurulent ocular discharge	Severe erosive/ulcerative lesions throughout buccal cavity, nasal mucosa and nares; oedematous lips and erosions on vulval labia	Muco-haemorrhagic diarrhoea	Marked tachypnoea / dyspnoea/ cough

End point definition: When animal reaches a score of 20 they need to be killed on ethical grounds. The decision to euthanasia would additionally be based in the following criteria: 1) A score of 4 is achieved in „General signs"; 2) A score of 3 is achieved in „General signs" for 2 complete, consecutive days and score of 10 or greater is achieved in other categories; 3) A score of 2 is achieved in „General signs" for 2 complete, consecutive days and score of 15 or greater is achieved in other categories. CS criteria in italic were modified from the CS sheet published by Pope et al. [2] according to Fowler [3] (* body temperatures given for adult SAC / D in moderate environment).

Table S2a. Literature overview of animal trials with cattle, buffaloes and camels experimentally infected with peste-des-petits-ruminants virus (PPRV) and with camels experimentally infected with rinderpest virus (RPV) (sorted by species, author and year of publication).

Common species name as given in reference (species)	Study design / inoculation route	Virus lineage (strain)	Virus antibodies in serum by no. of animals (method)	Virus RNA or antigen by matrix and no. of animals (method)	Virus isolation	Short description of report (including clinical signs)	Reference
Cattle experimentally infected with PPRV							
Cattle	s.c. infection of 3 cattle<br><br>s.c. infection of 3 cattle<br><br>s.c. infection of 3 cattle<br><br>s.c. infection of 3 cattle	PPRV LI (CIV89)<br><br>PPRV LII (Nigeria 75/3)<br><br>PPRV LIII (Ethiopia)<br><br>PPRV LIV (India-Calcutta)	3/3 (cELISA)<br><br>2/3 (cELISA)<br><br>3/3 (cELISA)<br><br>3/3 (cELISA)	swabs, blood: 0/3 (RT-qPCR)<br>swabs, blood: 0/3 (RT-qPCR)<br>swabs, blood: 0/3 (RT-qPCR)<br>swabs, blood: 0/3 (RT-qPCR)	NA<br><br>NA<br><br>NA<br><br>NA	After s.c. infection of cattle with PPRV of any one of the four PPRV lineages, cattle showed no clinical signs, no PPRV-RNA in blood and nasal and ocular swabs (0 to 30 dpi), no transmission to contact goats and sheep. Seroconversion in 11 of 12 animals. High antibody levels and seroconversion in all except 1 cattle that was infected with "mild" Nigeria 75/3 PPRV LII strain	Couacy-Hymann et al., 2019 [4]
Cattle	- contact infection of 3 calves by four experimentally PPRV-infected goats<br>-s.c. infection of 2 calves (10% spleen suspension) from PPRV-infected goat	PPRV LIV (EF641263, EF641264)	5/5 (cELISA)	-PBMCs: 5/5<br>-Nasal, oral and rectal swabs: 0/5 (RT-qPCR, sELISA)	isolation in B95a cells from PBMCs at 21 dpi (5/5, but 1 animal in each of 2 trials inconclusive), demonstration with IFAT and Vero cells; but no PPRV-RNA/-antigen excretion in swabs	subclinical PPRV-infection in 5 calves. PPRV isolation from PBMCs at 21 dpi, detection of antigen & RNA 21 to 397 dpi in PBMCs, but not in nasal, oral and rectal swabs tested 0 to 14 dpi	Sen et al., 2014 [5]

Common species name as given in reference (species)	Study design / inoculation route	Virus lineage (strain)	Virus antibodies in serum by no. of animals (method)	Virus RNA or antigen by matrix and no. of animals (method)	Virus isolation	Short description of report (including clinical signs)	Reference

Table S2a continued

Cattle	Infection with organ suspension from PPRV-infected goats (inoculation route NA)	PPRV	NA			After PPRV-infection of 6 calves, 4 calves were resistant to disease and 2 calves showed clinical signs after 6 or 8 dpi and succumbed disease or were sacrificed due to severe clinical signs at 11 or 13 dpi. Clinical signs included lacrimation, poor general condition, erosions or inflammations of upper and lower digestive tract, but PPRV was not considered the cause of the disease.	Mornet et al., 1956 [6]
Buffaloes experimentally infected with PPRV							
Indian buffalo (Bubalus bubalis)	s.c. inoculation of 2 Murrah buffalo calves with pool of mesenterial lymph node/spleen homogenate of a buffalo	PPR Ind TN 95/10 (96% sequence homology with African vaccine virus PPRV LII strain Nig 75/1)	NA	pool of mesenterial lymph node/spleen from each calve positive by RT-PCR	pool of mesenterial lymph node/spleen from each calve induced morbillivirus-like CPE in Vero cells	A PPRV isolate was obtained from a buffalo during an RPV outbreak in buffaloes in Tamil Nadu, India (see Table S3c). The 2 experimentally infected Murrah buffalo calves showed fever 3 to 6 dpi and died on 30 and 35 dpi. Necropsy revealed hemorrhagic and edematous abomastitis and gastroenteritis.	Govindarajan et al., 1997 [7]
Camels experimentally infected with PPRV							
Dromedary camel (*Camelus dromedarius*)	i.v. inoculation of five 2-year-old dromedaries with virulent PPRV LIV isolate	PPRV LIV (PPRV/MOR/2015)	5/5 (ELISA: AU-PANVAC, epitope-blocking ELISA with anti-H protein antibodies [8] and NT)	-blood: 0/5<br>-nasal, ocular and rectal swab samples: 0/5 (PCR)	NA	Five dromedary camels were i.v. infected with virulent PPRV LIV strain isolated in Morocco 2015. The used strain causes death in goats after 7-10 dpi with characteristic symptoms. No clinical signs detected in dromedaries during 6 weeks except slight transient congestion of ocular mucosa. no change in general behavior. No PPRV in blood and swab samples. 3/0 animals seropositive 14dpi, 5/5 positive 28 dpi by NT and ELISA. average NAbs 2.22 log10 in	Fakri et al., 2018 [9]

Table S2a continued

Common species name as given in reference (species)	Study design / inoculation route	Virus lineage (strain)	Virus antibodies in serum by no. of animals (method)	Virus RNA or antigen by matrix and no. of animals (method)	Virus isolation	Short description of report (including clinical signs)	Reference
						2 animals and 1.02 log10 in 3 animals at 42 dpi. Low antibody levels in camels compared to small ruminants. Camels seroconverted but did not shed or transmit the virus. Antibody levels considered low compared to small ruminants.	
Dromedary camel	s.c. inoculation (one 10-year-old pregnant)<br><br>i.v. inoculation (two 14-year-old)	PPRV LIII (Ethiopia 1994) (2ml of 10^5 TCID50/ml Vero cell culture virus)<br><br>PPRV LIII (Ethiopia 1994) (2ml of 10^5 TCID50/ml Vero cell culture virus)	2/3 (cELISA)	Blood: 0/3 (PCR)		Dromedaries were s.c. inoculated with PPRV LIII field or vaccine strain or antigen positive PPRV LIV strain. No clinical signs or increase in body temperature, no alteration in hematology observed. Pregnant camel did not abort and blood samples all negative in PCR. PPRV did not produce disease in dromedaries. Attenuated sheep PPRV vaccine did not induce seroconversion.	Wernery, 2011 [10]
	s.c. vaccination of five dromedaries with attenuated vaccine	PPRV LIII Jordanian sheep strain (1 ml of the vaccine Pestevac, Jovac, Jordan)	1/5 (cELISA)				
	s.c. inoculation of 5 ml supernatant of PPRV-antigen positive goat lung (one12-year-old male)	PPRV LIV	0/1 (cELISA)				

Common species name as given in reference (species)	Study design / inoculation route	Virus lineage (strain)	Virus antibodies in serum by no. of animals (method)	Virus RNA or antigen by matrix and no. of animals (method)	Virus isolation	Short description of report (including clinical signs)	Reference

Table S2a continued

Camel	Transmission study; experimental infection (unknown infection route) or field infection of 50 camels (6 months to 2 years old) with anyone of PPRV LI, LII, LIII, LIV; contact-controls were 50 goats and 50 sheep	PPRV LI, LII, LIII?, LIV	NA	RNA in blood (PCR, sequencing)<br>-camels<br>1st analysis: LI=8, LII=2;<br>2nd analysis: LI=12, LII=3, LIV=2.<br>-goats:<br>1st analysis: none<br>2nd analysis: LI=35<br>-of 50 sheep: none	Isolation from swabs<br><br>-camels<br>1st analysis: n=6,<br>2nd analysis: n=11.<br><br>-goats<br>1st analysis: negative<br>2nd analysis: n=32	50 camels were experimentally or naturally infected (no information) with different PPRV lineages (LI-LIV). Information about LIII infection is missing. Nasal and ocular swab samples were used for virus isolation but without further genotyping. Detection of PPRV-RNA of LI, LII and LIV in blood depending on time after infection. The time of sample collection and if the same animals were positive in the 1st and 2nd analysis is not clear. Samples were taken one month apart. Infection of 35 goats possibly by contact with camels infected with LI but it remains unclear if natural PPRV infection of goats (and camels) can be precluded.<br>Some (4 of 50) camels showed mild clinical signs and some no clinical signs, independent from the detection or isolation of PPRV. Results in goats were similar (21 of 50 show severe clinic, but 35 were PCR-positive). Fifty sheep remained PPRV-RNA negative and showed no clinical signs.	El-Hakim, 2006 [11]

Camels experimentally infected with RPV

Camel	i.v. infection with Kabete "O"	RPV Kabete "O"	1/1 after 14 dpi (NT)		1/1 viremia (isolation in steers but not in BK cells)	No transmission, no obvious clinical signs.	Taylor, 1968 [12]
	s.c. RPV infection	RPV RGK/I	2/2 after 10 or 18 dpi (NT)		1/2 viremia from 3 to 9 dpi (NA)	The camel that showed viremia 3-9 dpi had fever 7-9 dpi. No other clinical signs in the 2 infected camels. No transmission to 2 contact cattle and 1 contact camel.	

Common species name as given in reference (species)	Study design / inoculation route	Virus lineage (strain)	Virus antibodies in serum by no. of animals (method)	Virus RNA or antigen by matrix and no. of animals (method)	Virus isolation	Short description of report (including clinical signs)	Reference

Table S2a continued

	contact transmission from RPV-infected cattle to camels	RPV RGK/I	3/3 (after 16, 20 or 22 dpi) (NT)		Viremia 16 to 18 dpi in 2/3 contact infected camels (isolation in BK cells and subsequently in steers)	viremia in camels lower than in cattle but antibody titers similar. Contact infection of camels by RPV-infected cattle.	
Camel	s.c. experimental infection of 1 to 2-year-old local breed camels with any of two different RPV	RPV virulent Lybian 1966 or laboratory maintained Egyptian 1903 strain	2/2, high level seroconversion after 14 dpi (NT in BKC)			Fever for 1-2 days in camels but no other signs, no transmission to contact cattle	Singh and Ata, 1967 [13]
	vaccination of 4 camels (1 to 2-year-old) with one attenuated vaccine strain	RPV vaccine strain Kabete "O"	2/4, poor serological response after 14 or 28 dpi (NT in BKC)			Camels subclinically RPV-infected, high antibody titers after infection with two virulent strains, but low or no antibody titers using attenuated tissue culture RPV. no transmission of RPV from camel to calves. Only clinical sign transient fever. Field study: 8-10% camels of enzootic areas develop antibodies	

s.c., subcutaneous; i.v., intravenous; cELISA, competition ELISA; NT, neutralization test; NA, not available; RT-qPCR, real-time reverse transcription-PCR; sELISA, sandwich (antigen) ELISA; IFAT, indirect fluorescent antibody test; BK cells, primary calf kidney cells; BKC, bovine kidney cells; PBMCs, peripheral blood mononuclear cells; CPE, cytopathic effect; LI to LIV, PPRV lineages 1 to 4

Table S2b. Literature overview of field studies with cattle, buffaloes and camels naturally infected with peste-des-petits-ruminants virus (PPRV) and with camels naturally infected with rinderpest virus (RPV) (sorted by author and year of publication).

Common species name as given in reference (species)	Field study location (year)	Virus lineage (strain)	Virus antibodies in serum by prevalence (%); no. of animals (method)	Virus RNA or antigen by matrix and prevalence (%); no. of animals (method)	Virus isolation by matrix and prevalence (%); no. of animals (method)	Short description of report (including clinical signs)	Reference
Field studies of PPRV-infection in camels, cattle and/or buffaloes							
Dromedary camel	Study in 4 regions, Kenya	PPRV LIII, Kenya_PPRV_Camel_Mandera (60.29% nucleotide identity of camel and goat strains - two different regions)		1/25, (PCR) (sample matrix not given)		field study of clinical diseased camels (392 from 36 herds) mostly only clinical assessement. Detection of PPRV RNA in one sample of one campele (not given what kind of specimen - oral, nadsal swab or blood), same for the one of 7 goats, none of samples positive of the 4 sheep	Omani et al., 2019 [14]
Cattle	Marmara region, Turkey, field samples<br><br>Marmara region, Turkey, slaughterhouse or field samples	PPRV	0%; 0/50 (cELISA)	blood and nasal swabs: 0%; 0/50 (RT-qPCR)<br><br>lung homogenates: 0%; 0/50 (RT-qPCR)		PPR like clinical signs in 59.45% (66/111) sheep in the field and 48% (48/11) sheep with pneumonia or haemorrhages in lungs in slaughterhouse. PPRV LIV in found sheep: 11.7% of 111 seropositive, PPRV-RNA in 10.4% (22/211) lungs, swab or blood, PPRV-isolation from 2 sheep lungs. In cattle, no clinical signs, seroconversion or PPPRV-RNA detected despite of sample collection near the Bulgarian border where a PPRV outbreak occurred in small ruminants in 2018	Altan et al., 2018 [15]

Common species name as given in reference (species)	Field study location (year)	Virus lineage (strain)	Virus antibodies in serum by prevalence (%); no. of animals (method)	Virus RNA or antigen by matrix and prevalence (%) no. of animals (method)	Virus isolation by matrix and prevalence (%); no. of animals (method)	Short description of report (including clinical signs)	Reference

Table S2b continued

Cattle<br><br><br><br><br><br><br><br>Buffalo	Punjab province, Pakistan		10.0%; 24/240 (anti-hemagglutinin (H) PPRV competitive ELISA (cH-ELISA, BDSL, UK)<br>14.16%; 34/240 (cH-ELISA)			This focus of the study was the Punjab province where PPRV is endemic and where mixed farming practices<br>occur enabling close interactions between small and large ruminant populations. A higher seroprevalence was found in cattle (17.5%) and buffalo (22.5%) >2 years of age.	Abubakar et al., 2017 [16]
Cattle<br><br>Camel	Sudan		25.8%; 387/1501 (cELISA)<br>2.1%; 41/1988 (cELISA)	lung samples: 12.3% of 324 (IcELISA)<br>lung samples: 33.6% of 220 (IcELISA)	none tested<br><br>lung samples: 5/10 PPRV isolated	12,384 serum samples were collected from clinically healthy sheep, camels, cattle, goats and gazelles at different areas in Sudan Overall seroprevalence (cELISA) 49.4% (6112/12384). Seroprevalence in goats 48.2% (703/1459), sheep 67.1% (4976/7413) and gazelles 21.7% (5/23). Antigen (IcELISA) in 18.3% (233/1276) lung tissue samples collected from clinically healthy animals that showed lesions on PM in slaughterhouse (95%) and during PPR outbreaks. No lungs without lesions were tested. PPRV isolation from 15 of 30 antigen-positive (IcELISA) lung samples. Virus isolation with primary bovine and ovine kidney cells and Vero cells. Antigen prevalence in camels (33.6%) was	Intisar et al., 2017 [17]

Common species name as given in reference (species)	Field study location (year)	Virus lineage (strain)	Virus antibodies in serum by prevalence (%); no. of animals (method)	Virus RNA or antigen by matrix and prevalence (%) no. of animals (method)	Virus isolation by matrix and prevalence (%); no. of animals (method)	Short description of report (including clinical signs)	Reference

Table S2b continued

						similar to goats (21.1% of 109 lungs), but seroprevalence was considerably lower in camels (2.1% vs 48.2%). Antigen in 15.4% of 623 sheep lungs.	
Camel	Khuzestan province, Iran (2013)	PPRV LIV (99% homology by Iranian sub-strain DQ840185) negative for bacteria	NA	PPRV antigen RNA and sequencing: in tissue of 2 dead camels. Histopathology without IHC or ISH (no details about organs)	NA (no data, not clear if "tissue culture isolate" means RNA or infectious virus)	Mid-July 2013 outbreak fatal disease in camel herd two weeks after import from Kuwait. 30 camels with clinical signs, 12 deaths within 14 days. Leukocytopenia and lymphopenia. No details on seroprevalence and of PPRV RNA or antigen in organs. IcELISA confirmed presence of antigen in tissue samples from infected camels. RT-PCR detected PPRV-RNA in tissue. Sequencing revealed 99% homology by Iranian sub-strain. Two weeks after importing camels clinical disease occurred in small ruminant flocks. Clinical signs of the affected camels after import from Kuwait included sudden death, fever, oral erosion, and ecthyma like lesions, yellowish diarrhea, pneumonia and respiratory distress, enlargement of lymph node, severe dehydration, dermatitis, ulcerative keratitis, and conjunctivitis. Necropsy findings	Zakian et al., 2016 [18]

Common species name as given in reference (species)	Field study location (year)	Virus lineage (strain)	Virus antibodies in serum by prevalence (%); no. of animals (method)	Virus RNA or antigen by matrix and prevalence (%) no. of animals (method)	Virus isolation by matrix and prevalence (%); no. of animals (method)	Short description of report (including clinical signs)	Reference

Table S2b continued

						included keratoconjunctivitis, congestion and consolidation of the lung, paleness of the liver, and enlargement and edema of lymph nodes. Histopathological exam revealed degeneration and acute hyperemia of the lungs, fatty change and necrotic foci in the liver, tubular necrosis in the kidneys, and necrotic dermatitis	
Dromedary camel	Study in 5 areas, Sudan			lung samples: 45.1%; 214/474 (IcELISA)		Field study of mixed infections in lung samples of dromedaries. Mixed infections with other respiratory viruses (PIV3, RSV, BVDV, BHV-1, adenoviruses) in 32 of 214 PPRV-antigen positive lung samples from clinically healthy camels with lungs that showed pneumonia in slaughterhouses. Results revealed the existence of PPRV in dromedary and present evidence for mixed virus infection, suggesting that respiratory infections in camels might be exacerbate by PPRV. Most frequenly noticed mixed infections: 9 x PPRV/BVDV, 6 x PPRV/PIV3, 6 x PPRV/RSV. No lungs without pneumonia were tested.	Saeed et al., 2015 [19]

Common species name as given in reference (species)	Field study location (year)	Virus lineage (strain)	Virus antibodies in serum by prevalence (%); no. of animals (method)	Virus RNA or antigen by matrix and prevalence (%) no. of animals (method)	Virus isolation by matrix and prevalence (%); no. of animals (method)	Short description of report (including clinical signs)	Reference

Table S2b continued

Camel	Study in 4 states of Nigeria		3.36%; 51/1517 (anti-H cELISA)			Seroprevalence in 4 states of Nigeria. No significant differences in prevalences between 4 states and male and females. Prevalence differed significantly by body condition score (poor 16.67, fair 3.43, good 2.39%). Significant differences by age (+/- 5 years, higher in younger animals).	Woma et al., 2015 [20]
Cattle<br><br><br><br>Buffalo	Study in 52 districts, India (2011)		11.07%; 67/605 (in-house cELISA)<br><br>16.20%; 70/432 (in-house cELISA)			Overall seroprevalence 21.83%. Seroprevalence in sheep 45.66% (79/173) and goats 38.54% (11/288). Similar seroprevalence between large ruminants and between small ruminants. Bovines subclinically infected. High prevalence in bovines may be due to random samples collected from villages where cattle, buffaloes, sheep and goats are reared together (epidemiological unit).	Balamurugan et al., 2014 [21]
Cattle	Tanzania (<2008-2012)	PPRV	17.3%; 46/266, locally 7 to 48% (cELISA, anti-H, Biological Diagnostic, BDSL, UK; results confirmed			no clinic described /available; study samples from before 2008 and 2008 to 2012; 26.7% of the samples from cattle that were alive during the 2008 PPR outbreak were seropositive, and 5.9% from cattle born after the outbreak. Seroprevalence in village cattle ranged from 7% to 48%. Serum	Lembo et al., 2013 [22]

Common species name as given in reference (species)	Field study location (year)	Virus lineage (strain)	Virus antibodies in serum by prevalence (%); no. of animals (method)	Virus RNA or antigen by matrix and prevalence (%) no. of animals (method)	Virus isolation by matrix and prevalence (%); no. of animals (method)	Short description of report (including clinical signs)	Reference

Table S2b continued

African buffalo (*Syncerus caffer*)			with cELISA anti-N, IDvet)<br><br>0%; 0/266 (anti-H and anti-N cELISA)			samples from African buffaloes came from an archived serum bank. Some samples could be dated to 2011-2012.	
Camel	Study in 5 regions in Sudan (2000-2009)	PPRV LIV		lung, liver, spleen: 77.6%; 38 of 49, PPRV-RNA (RT-PCR and sequencing)	PPRV isolation from lung samples of 3 camels: Cam_8, Cam_169, Cam_318 (MDBK cells)	80 field samples collected from animals with PPRV-like clinical signs in 5 regions of Sudan (2000-2009). In total, 64/80 animals positive by PCR. All 5 goats and 80.8% (21 of 26) sheep PCR-positive lung, liver, spleen. PPRV LIV defined by 2 subclusters (one cluster with camel and goat and some sheep isolates, other cluster only with sheep isolates - but may be second cluster from morocco). PPRV isolation from 2 sheep lungs and 3 camel lungs.	Kwiatek et al., 2011 [23]
Camel	Sudan	PPRV LIV (cited Kwiatek et al. 2011)		lungs, ln.: 6/6 (AGPT, IcELISA), 5/6 (RT-PCR)	One sample CPE after 3 passages on MDBK cells. PPRV confirmed with AGPT,	Study of death in camels during a PPR outbreak in Sudan.	Khalafalla et al., 2010 [24]

Common species name as given in reference (species)	Field study location (year)	Virus lineage (strain)	Virus antibodies in serum by prevalence (%); no. of animals (method)	Virus RNA or antigen by matrix and prevalence (%) no. of animals (method)	Virus isolation by matrix and prevalence (%); no. of animals (method)	Short description of report (including clinical signs)	Reference

Table S2b continued

					ELISA, RT-PCR.		
Cattle<br><br>Dromedary camel (*Camelus dromedaries*)	Turkey, Aydin province	PPRV	18%; 22/122 (cELISA, Biological Diagnostic, UK)<br><br>0%; 0/18 (cELISA, Biological Diagnostic, UK)	NA	NA	PPR-related fatalities in sheep (12.3%; 16/130) one month before the field study. Seroprevalence in sheep 88% (44/50). No clinical signs in cattle and camels. Cattle from dairy herds 1-6 years old (no vaccination against PPRV or RPV). Only a few camels left in Turkey, which are mainly used for races and breeding indoor. Therefore, no contact grazing of camels with other ruminant species	Albayrak and Gür, 2010 [25]
Cattle<br><br>Buffalo	Punjab province, Pakistan	PPRV	41.86%; 18/43 (cELISA)<br><br>67.42%; 60/89 (cELISA)			Field study without analysis of clinical signs. Seroprevalence in sheep 51.29% (119/232) and goats 39.02% (167/428). Reason for high seroprevalence in large ruminants explained by common grazing grounds with small ruminants. High seroprevalence in sheep explained due to higher recovery rate than in goats.	Khan et al., 2008 [26]
Dromedary camel	Dubai, UAE, in milk producing herd	PPRV	0% of 1119 (cELISA)			Seroepidemiological studies for the detection of antibodies against 9 different infectious diseases in dairy dromedaries. 541 milking camels and 578 of their calves, belonging to the "Emirates	Wernery et al., 2007 [27]

Common species name as given in reference (species)	Field study location (year)	Virus lineage (strain)	Virus antibodies in serum by prevalence (%); no. of animals (method)	Virus RNA or antigen by matrix and prevalence (%) no. of animals (method)	Virus isolation by matrix and prevalence (%); no. of animals (method)	Short description of report (including clinical signs)	Reference

Table S2b continued

						Industry for Camel Milk and Products" in Dubai.	
Cattle<br><br>Camel	Ethiopia (2001)	PPRV	9% of 910, locally up to 16% (cELISA)<br><br>3% of 628, locally up to 10% (cELISA)			Respiratory disease observed in camels, goats, sheep, but not clear whether PPRV was cause of respiratory disease. Seroprevalence in sheep 13% of 835 and goats 9 % of 442 (locally up to 22% respectively 23%). Higher seroprevalence found in cattle than in camels was explained by a higher population density and mixed grazing resulting in increased contact between small ruminants and cattle.	Abraham et al., 2005 [28]
Cattle<br><br>Camel	Sudan	PPRV and RPV (RPV all seronegative)	11.4%; 4/35 (ELISA)<br><br>14%; 14/100 (ELISA)			Serosurveillance study in Sudan. Seroprevalence in sheep 51.9% (27/52) and goats 56.2% (27/48)	Haroun et al., 2002 [29]
Camel	Ethiopia	PPRV; PPRV LII (Ethiopia 96 CAMEL 1), LIII (Ethiopia 96 CAMEL 2)<br>Co-infection with Streptococcus equi subsp. equi isolated (but no pasteurella)	yes	Yes, RNA (RT-PCR, sequencing), antigen (ELISA)		Epizootic disease in Ethiopia with high morbidity (90%) and mortality rates (5 to 70%) in camel. May 1995 to September 1996. Detection of antibodies, antigen and RNA but no numbers given. Sequences of 2 strains of "morbillivirus" from PPRV LII and LIII closely related to ovine strains. Virus	Roger et al., 2000 [30]

Common species name as given in reference (species)	Field study location (year)	Virus lineage (strain)	Virus antibodies in serum by prevalence (%); no. of animals (method)	Virus RNA or antigen by matrix and prevalence (%) no. of animals (method)	Virus isolation by matrix and prevalence (%); no. of animals (method)	Short description of report (including clinical signs)	Reference

Table S2b continued

						immunosuppression might have favored pathogenicity of secondary bacterial infection already treated by antibiotics. Streptococcus equi subsp. equi isolated from camels (but no pasteurella)	
Indian buffalo (*Bulbalus bubalis*)	Tamil Nadu, India	PPRV, (RPV) PPR Ind TN 95/10 (96% sequence homology with African vaccine virus PPR Nig 75/1)		2 sets of mesenteric ln. and spleen positive for PPRV antigen; thereof 1 pool positive by RT-PCR and sequencing	2 sets of mesenteric ln. and spleen showed CPE in Vero cells	Sample collection during an RPV outbreak. Clinical RP in 13% (50/385) buffaloes thereof 6 of 10 tissue (mesenteric ln., spleen) RPV-antigen positive. Likely 2 of 385 buffaloes positive for PPRV. One pool positive by PCR, sequencing (PPR Ind TN 95/10) and isolation used for experimental infection of buffalo calves (see table S3a).	Govindarajan et al., 1997 [7]

Field studies of RPV-infection in camels

Camel	Egypt, slaughterhouse	RPV	9.2%; 9/97 (NT with BKC cultures)			Of 97 camels (25 local Egypt and 72 imported from Sudan) 9.2% (9/97), 8% (2/25) and 9.7% (7/72) seropositive for RPV	Singh and Ata, 1967 [13]
Camel	Kenya	RPV		0/60 (NT using 3 rabbits per sample)		None of 60 camels sampled during RP outbreak in cattle were RPV seropositive. Different reports of RPV field studies in camels are discussed. Based on clinical signs and coinicidental RPV outbreaks in cattle controversial results were found for camels. No proof that camels show clinical signs to RPV infection.	Scott and MacDonald, 1962 [31]

Table S3b continued

s.c., subcutaneous; i.v., intravenous; cELISA, competition ELISA; NT, neutralization test; NA, not available; RT-qPCR, real-time reverse transcription-PCR; IcELISA, immunocapture ELISA; AGPT, agar gel precipitation test; BKC, bovine kidney cell culture; PBMCs, peripheral blood mononuclear cells; CPE, cytopathic effect; LI to LIV, PPRV lineages 1 to 4; ln, lymph node

Table S3. Statistical results of Cq values obtained from swab samples collected from different Artiodactyla species over time during transmission trials. Oronasal, conjunctival and fecal swabs were collected from animals of different Artiodactyla species after experimental intranasal infection or contact infection with PPRV lineage IV strain Kurdistan/2011 and analyzed by PPRV-specific RT-qPCR. The data used from goats, pigs, wild boar and sheep were collected in transmission trials published previously [32]. The goodness of fit of Cq values of the swab samples tested by animal species with Shapiro-Wilk normality test revealed no normal distribution. Accordingly, p-values were calculated using i) a linear mixed-effects (lme) model including random effects (individual animal) and fixed effects (animal species and days after infection as continuous variables) (lower left triangle of table) and ii) independent 2-group Mann-Whitney test with Bonferroni correction (upper right triangle of table) using R software (www.r-project.org; packages stats and nlme). Significant differences were found between PPRV-RNA amounts over time in all swab samples from goats in comparison to the other artiodactyls, independent from the statistical method used. Goats excreted significantly higher PPRV-RNA amounts over time compared to the other artiodactyls, independent from the swab material and the method used. SAC generally excreted the significantly lowest amounts of PPRV-RNA over time compared with the other artiodactyls. In contrast, PPRV-RNA loads in oronasal and conjunctival swab samples were not significantly different between cattle, wild boar and pigs, respectively. Fecal swab samples from cattle contained significantly lower PPRV-RNA loads compared with other artiodactyls, except for SAC fecal swab samples using the lme model.

swab material	animal spp. (no.)	goat (n=4)	cattle (n=3)	pig (n=5)	SAC (n=6)	sheep (n=5)	wild boar (n=4)
oronasal	goat	30.725	0.000	0.015	0.000	0.013	0.000
	cattle	0.000	40.290	0.000	0.000	0.001	**0.322**
	pig	0.016	0.001	34.717	0.000	**0.885**	0.000
	SAC	0.000	0.034	0.000	43.227	0.000	0.000
	sheep	0.008	0.002	**0.675**	0.000	35.271	0.005
	wild boar	0.000	**0.381**	0.003	0.002	0.008	39.062
conjunctival	goat	31.971	0.000	0.000	0.000	0.014	0.000
	cattle	0.000	42.383	**0.052**	0.000	0.000	**0.705**
	pig	0.000	**0.192**	40.037	0.000	0.012	0.009
	SAC	0.000	**0.093**	0.002	45.230	0.000	0.000
	sheep	0.013	0.004	**0.051**	0.000	36.781	0.000
	wild boar	0.000	**0.822**	**0.100**	**0.109**	0.001	42.786
fecal	goat	31.137	0.000	0.000	0.000	0.001	0.001
	cattle	0.000	44.462	0.000	0.010	0.000	0.000
	pig	0.000	0.000	38.141	0.000	**0.388**	**0.978**
	SAC	0.000	**0.472**	0.000	45.372	0.000	0.000
	sheep	0.000	0.000	**0.581**	0.000	37.431	**0.614**
	wild boar	0.000	0.000	**0.897**	0.000	**0.492**	38.302

Intercept Cq values for different animal spp. as calculated by lme are highlighted in grey. P-values of alpha < 0.05 were considered statistically significant. Non-significant p-values are highlighted in **bold** and p-values found non-significant in both statistical tests are additionally highlighted in **red**.

Supplemental Materials References

1. Stober, M. Kennzeichen, Anamnese, Grundregeln der Untersuchungstechnik, Allgemeine Untersuchung. In *Die klinische Untersuchung des Rindes*, 3 ed.; Rosenberger, G., Ed. Parey: Berlin, Hamburg, 1990; Vol. 3rd ed., pp. 75-141.
2. Pope, R.A.; Parida, S.; Bailey, D.; Brownlie, J.; Barrett, T.; Banyard, A.C. Early events following experimental infection with Peste-Des-Petits ruminants virus suggest immune cell targeting. *PLoS One* **2013**, *8*, e55830, doi:10.1371/journal.pone.0055830.
3. Fowler, M.E. Clinical Diagnosis: Examination and Procedures. In *Medicine and Surgery of Camelids*, Fowler, M.E., Ed. Blackwell Publishing: Ames, IA, 2010; Vol. 3rd, pp. 89-109.
4. Couacy-Hymann, E.; Koffi, M.Y.; Kouadio, V.K.; Mossoum, A.; Kouadio, L.; Kouassi, A.; Assemian, K.; Godji, P.H.; Nana, P. Experimental infection of cattle with wild type peste-des-petits-ruminants virus - Their role in its maintenance and spread. *Res. Vet. Sci.* **2019**, *124*, 118-122, doi:10.1016/j.rvsc.2019.02.011.
5. Sen, A.; Saravanan, P.; Balamurugan, V.; Bhanuprakash, V.; Venkatesan, G.; Sarkar, J.; Rajak, K.K.; Ahuja, A.; Yadav, V.; Sudhakar, S.B., et al. Detection of subclinical peste des petits ruminants virus infection in experimental cattle. *Virusdisease* **2014**, *25*, 408-411, doi:10.1007/s13337-014-0213-0.
6. Mornet, P.; Gilbert, Y.; Orue, J.; Thiéry, G. La peste des petits ruminants en Afrique occidentale française. Ses rapports avec la peste bovine. *Revue d'élevage et de médecine vétérinaire des pays tropicaux* **1956**, *9*, 313-342.
7. Govindarajan, R.; Koteeswaran, A.; Venugopalan, A.T.; Shyam, G.; Shaouna, S.; Shaila, M.S.; Ramachandran, S. Isolation of pestes des petits ruminants virus from an outbreak in Indian buffalo (*Bubalus bubalis*). *Vet. Rec.* **1997**, *141*, 573-574.
8. Bodjo, S.C.; Baziki, J.D.; Nwankpa, N.; Chitsungo, E.; Koffi, Y.M.; Couacy-Hymann, E.; Diop, M.; Gizaw, D.; Tajelser, I.B.A.; Lelenta, M., et al. Development and validation of an epitope-blocking ELISA using an anti-haemagglutinin monoclonal antibody for specific detection of antibodies in sheep and goat sera directed against peste des petits ruminants virus. *Arch. Virol.* **2018**, *163*, 1745-1756, doi:10.1007/s00705-018-3782-1.
9. Fakri, F.Z.; Bamouh, Z.; Jazouli, M.; Tadlaoui, K.; Elharrak, M. Experimental infection of dromedary camels with virulent virus of Peste des petits ruminants In Proceedings of The 5th Conference of the International Society of Camel Research and Development (ISOCARD).
10. Wernery, U. Peste des petits ruminants (PPR) in camelids with own investigations. *J Camel Practice Research* **2011**, *18*, 219-223.
11. El-Hakim, U.A. The role of camels in dessimination of peste des petits ruminants virus among sheep and goats in Saudi Arabia. *Assiut Vet. Med. J.* **2006**, *52*, 132-145.
12. Taylor, W.P. The susceptibility of the one-humped camel (*Camelus dromedarius*) to infection with rinderpest virus. *Bull. Epizoot. Dis. Afr.* **1968**, *16*, 405-410.
13. Singh, K.V.; Ata, F. Experimental rinderpest in camels. A preliminary report. *Bull Epizoot Dis Afr* **1967**, *15*, 19-23.
14. Omani, R.N.; Gitao, G.C.; Gachohi, J.; Gathumbi, P.K.; Bwihangane, B.A.; Abbey, K.; Chemweno, V.J. Peste Des Petits Ruminants (PPR) in Dromedary Camels and Small Ruminants in Mandera and Wajir Counties of Kenya. *Adv Virol* **2019**, *2019*, 4028720, doi:10.1155/2019/4028720.
15. Altan, E.; Parida, S.; Mahapatra, M.; Turan, N.; Yilmaz, H. Molecular characterization of Peste des petits ruminants viruses in the Marmara Region of Turkey. *Transbound. Emerg. Dis.* **2018**, 10.1111/tbed.13095, doi:10.1111/tbed.13095.
16. Abubakar, M.; Mahapatra, M.; Muniraju, M.; Arshed, M.J.; Khan, E.H.; Banyard, A.C.; Ali, Q.; Parida, S. Serological Detection of Antibodies to Peste des Petits Ruminants Virus in Large Ruminants. *Transbound Emerg Dis* **2017**, *64*, 513-519, doi:10.1111/tbed.12392.
17. Intisar, K.S.; Ali, Y.H.; Haj, M.A.; Sahar, M.A.; Shaza, M.M.; Baraa, A.M.; Ishag, O.M.; Nouri, Y.M.; Taha, K.M.; Nada, E.M., et al. Peste des petits ruminants infection in domestic ruminants in Sudan. *Trop. Anim. Health. Prod.* **2017**, *49*, 747-754, doi:10.1007/s11250-017-1254-3.
18. Zakian, A.; Nouri, M.; Kahroba, H.; Mohammadian, B.; Mokhber-Dezfouli, M.R. The first report of peste des petits ruminants (PPR) in camels (Camelus dromedarius) in Iran. *Trop Anim Health Prod* **2016**, *48*, 1215-1219, doi:10.1007/s11250-016-1078-6.
19. Saeed, I.K.; Ali, Y.H.; AbdulRahman, M.B.; Mohammed, Z.A.; Osman, H.M.; Taha, K.M.; Musa, M.Z.; Khalafalla, A.I. Mixed infection of peste des petits ruminants virus (PPRV) and other respiratory viruses in dromedary camels in Sudan, an abattoir study. *Trop Anim Health Prod* **2015**, *47*, 995-998, doi:10.1007/s11250-015-0798-3.
20. Woma, T.Y.; Kalla, D.J.; Ekong, P.S.; Ularamu, H.G.; Chollom, S.C.; Lamurde, II; Bajehson, D.B.; Tom, N.D.; Aaron, G.B.; Shamaki, D., et al. Serological evidence of camel exposure to peste des petits

ruminants virus (PPRV) in Nigeria. *Trop Anim Health Prod* **2015**, *47*, 603-606, doi:10.1007/s11250-014-0747-6.
21. Balamurugan, V.; Krishnamoorthy, P.; Raju, D.S.; Rajak, K.K.; Bhanuprakash, V.; Pandey, A.B.; Gajendragad, M.R.; Prabhudas, K.; Rahman, H. Prevalence of Peste-des-petits-ruminant virus antibodies in cattle, buffaloes, sheep and goats in India. *Virusdisease* **2014**, *25*, 85-90, doi:10.1007/s13337-013-0177-5.
22. Lembo, T.; Oura, C.; Parida, S.; Hoare, R.; Frost, L.; Fyumagwa, R.; Kivaria, F.; Chubwa, C.; Kock, R.; Cleaveland, S., et al. Peste des petits ruminants infection among cattle and wildlife in northern Tanzania. *Emerg Infect Dis* **2013**, *19*, 2037-2040, doi:10.3201/eid1912.130973.
23. Kwiatek, O.; Ali, Y.H.; Saeed, I.K.; Khalafalla, A.I.; Mohamed, O.I.; Obeida, A.A.; Abdelrahman, M.B.; Osman, H.M.; Taha, K.M.; Abbas, Z., et al. Asian lineage of peste des petits ruminants virus, Africa. *Emerg Infect Dis* **2011**, *17*, 1223-1231, doi:10.3201/eid1707.101216.
24. Khalafalla, A.I.; Saeed, I.K.; Ali, Y.H.; Abdurrahman, M.B.; Kwiatek, O.; Libeau, G.; Obeida, A.A.; Abbas, Z. An outbreak of peste des petits ruminants (PPR) in camels in the Sudan. *Acta Trop.* **2010**, *116*, 161-165, doi:10.1016/j.actatropica.2010.08.002.
25. Albayrak, H.; Gür, S. A serologic investigation for Peste des petits ruminants infection in sheep, cattle and camels (Camelus dromedarius) in Aydin province, West Anatolia. *Trop. Anim. Health. Prod.* **2010**, *42*, 151-153, doi:10.1007/s11250-009-9400-1.
26. Khan, H.A.; Siddique, M.; Sajjad ur, R.; Abubakar, M.; Ashraf, M. The detection of antibody against peste des petits ruminants virus in sheep, goats, cattle and buffaloes. *Trop. Anim. Health. Prod.* **2008**, *40*, 521-527, doi:10.1007/s11250-008-9129-2.
27. Wernery, U.; Thomas, R.; Syriac, G.; Raghavan, G.; Kletzka, S. Seroepidemiological studies for the detection of antibodies against nine infectious diseases in dairy dromedaries (Part-I). *J Camel Practice Research* **2007**, *14*, 85-90.
28. Abraham, G.; Sintayehu, A.; Libeau, G.; Albina, E.; Roger, F.; Laekemariam, Y.; Abayneh, D.; Awoke, K.M. Antibody seroprevalences against peste des petits ruminants (PPR) virus in camels, cattle, goats and sheep in Ethiopia. *Prev. Vet. Med.* **2005**, *70*, 51-57, doi:10.1016/j.prevetmed.2005.02.011.
29. Haroun, M.; Hajer, I.; Mukhtar, M.; Ali, B.E. Detection of antibodies against peste des petits ruminants virus in sera of cattle, camels, sheep and goats in Sudan. *Vet. Res. Commun.* **2002**, *26*, 537-541.
30. Roger, F.; Yigezu, L.M.; Hurard, C.; Libeau, G.; Mebratu, G.Y.; Diallo, A.; Faye, B. Investigations on a new pathological condition of camels in Ethiopia. *Journal of camel practice and research* **2000**, *7*, 163-165.
31. Scott, G.R.; MacDonald, J. Kenya camels and rinderpest. *Bull. epiz. Dis. Afr.* **1962**, *10*, 495-497.
32. Schulz, C.; Fast, C.; Schlottau, K.; Hoffmann, B.; Beer, M. Neglected hosts of small ruminant morbillivirus. *Emerg. Infect. Dis.* **2018**, *24*, 2334-2337, doi:10.3201/eid2412.180507.
